# Data mining approach identifies research priorities and data requirements for resolving the red algal tree of life

**DOI:** 10.1186/1471-2148-10-16

**Published:** 2010-01-20

**Authors:** Heroen Verbruggen, Christine A Maggs, Gary W Saunders, Line Le Gall, Hwan Su Yoon, Olivier De Clerck

**Affiliations:** 1 Phycology Research Group and Center for Molecular Phylogenetics and Evolution, Ghent University, Krijgslaan 281, building S8, 9000 Ghent, Belgium; 2 School of Biological Sciences, Queen's University Belfast, Medical Biology Centre, 97 Lisburn Road, Belfast BT9 7BL, UK; 3 Centre for Environmental & Molecular Algal Research Department of Biology, University of New Brunswick, Fredericton, NB E3B 5A3, Canada; 4 Muséum National d'Histoire Naturelle, UMR 7138, CP #39, 57 rue Cuvier, 75231 Cedex 05 Paris, France; 5 Bigelow Laboratory for Ocean Sciences, 180 McKown Point Road, West Boothbay Harbor, ME 04575, USA

## Abstract

**Background:**

The assembly of the tree of life has seen significant progress in recent years but algae and protists have been largely overlooked in this effort. Many groups of algae and protists have ancient roots and it is unclear how much data will be required to resolve their phylogenetic relationships for incorporation in the tree of life. The red algae, a group of primary photosynthetic eukaryotes of more than a billion years old, provide the earliest fossil evidence for eukaryotic multicellularity and sexual reproduction. Despite this evolutionary significance, their phylogenetic relationships are understudied. This study aims to infer a comprehensive red algal tree of life at the family level from a supermatrix containing data mined from GenBank. We aim to locate remaining regions of low support in the topology, evaluate their causes and estimate the amount of data required to resolve them.

**Results:**

Phylogenetic analysis of a supermatrix of 14 loci and 98 red algal families yielded the most complete red algal tree of life to date. Visualization of statistical support showed the presence of five poorly supported regions. Causes for low support were identified with statistics about the age of the region, data availability and node density, showing that poor support has different origins in different parts of the tree. Parametric simulation experiments yielded optimistic estimates of how much data will be needed to resolve the poorly supported regions (ca. 10^3 ^to ca. 10^4 ^nucleotides for the different regions). Nonparametric simulations gave a markedly more pessimistic image, some regions requiring more than 2.8 10^5 ^nucleotides or not achieving the desired level of support at all. The discrepancies between parametric and nonparametric simulations are discussed in light of our dataset and known attributes of both approaches.

**Conclusions:**

Our study takes the red algae one step closer to meaningful inclusion in the tree of life. In addition to the recovery of stable relationships, the recognition of five regions in need of further study is a significant outcome of this work. Based on our analyses of current availability and future requirements of data, we make clear recommendations for forthcoming research.

## Background

Several approaches can be taken to resolving the tree of life, the most effective often depending on the nature of the specific project and the availability of previously collected data. Whereas only one or a few loci are required to resolve the relationships among a set of recently diverged species, much larger amounts of comparative data are needed to reconstruct ancient branches of the tree of life. An important source of molecular data for probing deep into evolutionary time comes from genomic studies (whole genome sequences and EST libraries). For resolving branches of intermediate age, targeted PCR amplification and sequencing of multiple genes is often preferred. More often than not, some DNA data relevant to a given problem are available on public databases (e.g., GenBank) and not all projects require newly generated data. Mining data repositories to construct comprehensive phylogenetic trees is one of the foci of contemporary research [1-4].

During the past decade, major progress has been made in assembling the tree of life, using a range of approaches. At one end of this spectrum, genome-scale phylogenetics have been applied to resolve the ancient evolutionary relationships between the major groups of eukaryotes [5-7]. Such studies are based on large amounts of DNA data for a small set of species. At the other extreme, phylogenetic trees including almost all extant species have been assembled for some well-studied groups such as mammals [8]. Most projects, however, are situated in between these extremes and attempt to infer the relationships among representatives of families or orders based on a handful of loci [9,10].

It has been shown that the amount of data available to infer a phylogenetic tree will affect its accuracy and the statistical confidence in its branching pattern. Theoretical and empirical studies have shown that both the length of the sequence alignment and the number and selection of taxa are important in this respect [11-16]. If a large number of lineages diverged from each other in a short period of time, phylogenetic reconstruction becomes notoriously difficult because there has been little time for base substitutions to accumulate between the subsequent cladogenesis events and different genes are more likely to have discordant phylogenetic histories [17-19]. If such rapid radiations occurred in ancient times, phylogenetic reconstruction is further hindered because the signal about the radiation that was left in the DNA is more likely to be overwritten and masked by substitutions occurring during the long time span between the radiation and the present [19,20]. Complementary to the research into the effects of data availability on the accuracy of phylogenetic inference, various studies have attempted to estimate the amount of data needed to reconstruct difficult phylogenetic problems, most often using simulation approaches [21-26].

In general, the phylogenetic relationships among algae and other unicellular eukaryotes (protists) have been investigated in much less detail than those of more conspicuous organisms like birds, mammals and higher plants. The present study focuses on red algae, which were specifically listed as an under-studied group in the report of a recent workshop on the future of the NSF-sponsored AToL project [27]. The red algae or Rhodophyta form one of the three major lineages of primary photosynthetic organisms that evolved after the enslavement of a cyanobacterium in a eukaryote cell to form a chloroplast more than 1.5 billion years ago [28,29] and the earliest fossil evidence for multicellular eukaryotic life, *Bangiomorpha *from the 1,200 Ma Hunting formation, is thought to be a red alga [30].

The Rhodophyta contain the accessory pigments phycocyanin and phycoerythrin [31]. Their cytoplasm contains floridean starch grains and adjacent cells are linked by protoplasmic connections in which proteinaceous plugs are formed [32]. Perhaps the most striking red algal feature is the complete lack of 9 + 2 microtubule structures such as flagella and centrioles [32,33]. The red algae are currently credited with about 6000 species in ca. 700 genera [34]. They are mostly marine, with some freshwater genera and one class of volcano-loving extremophiles, the Cyanidiophyceae. The great majority of red algae are multicellular, with an enormous range of morphologies. Their life histories are complex and in the majority of lineages an additional zygote amplification stage results in large numbers of spores from a single fertilization.

Red algal systematics has seen many improvements over the past decades. Starting from a classification based on morphological and reproductive features half a century ago [35], a series of ultrastructural investigations and life-cycle analyses has progressively refined the ordinal classification. Over the past two decades, DNA sequence data has brought additional resolution to the higher-level classification. The earliest two attempts at reconstructing a red algal tree of life based on single genes (18S rDNA and *rbc*L) were published back-to-back in PNAS in 1994 [36,37] and indicated the paraphyly of the Bangiophyceae, which was confirmed and detailed in later work [38,39]. A series of single- and multi-gene phylogenetic studies by Saunders and co-workers provided increasingly detailed and taxonomically important overviews of relationships among florideophyte orders, culminating with the proposal of a series of new subclasses [40-44]. However, despite intensive effort, a lack of resolution of the relationships among florideophyte clades has remained and there is as yet no comprehensive phylogeny of the red algae.

The first goal of this study was to generate a comprehensive red algal tree of life at the family level based on currently available data. Our approach consists of mining the DNA data in GenBank to construct a supermatrix and analyzing this matrix with model-based phylogenetic inference techniques. Our second goal was to locate well- and poorly supported regions in the topology, evaluate the possible causes of the remaining poorly supported relationships, and formulate future research priorities based on this information. We approached this goal by identifying poorly supported regions with a simple visualization technique and calculating several statistics pertaining to data availability and the difficulty of resolving poorly supported regions. Finally, the amount of data needed to resolve the poorly supported regions is estimated with parametric and nonparametric simulation experiments.

## Results

### Dataset and model selection

**Figure 1:**
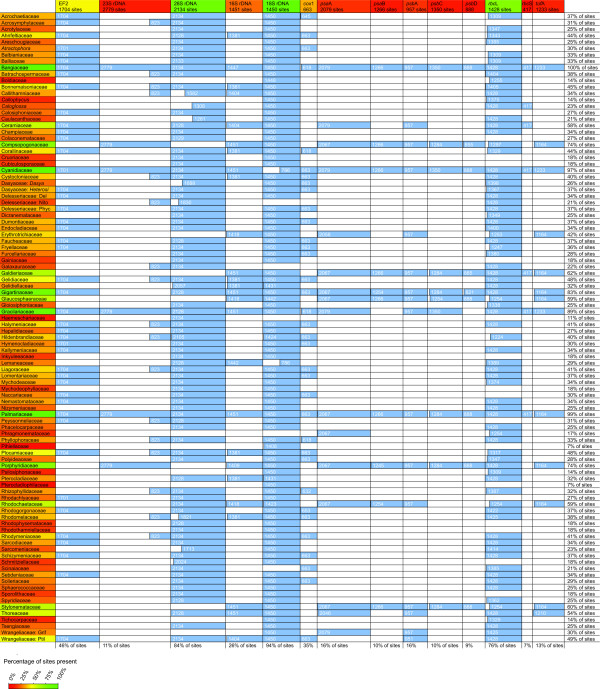
**Data availability matrix**. Graphical representation of our concatenated alignment, showing the availability of sequence data. The color of column and row headers indicate the amount of data available for that column or row. Green indicates high data availability, red indicates low data availability and yellow/orange represents intermediate data availability. The matrix density is 34% in a locus × OTU context and 35% in a character × OTU context. Numbers in cells indicate length of sequence in alignment, which may include gaps and/or exclude ambiguously aligned regions. Figure generated with the gDAM software http://www.phycoweb.net.

Data mined from GenBank in combination with a small number of new sequences allowed us to construct a supermatrix consisting of 98 OTUs and 14 loci (19,799 characters). The supermatrix was 34% complete in a locus × OTU context and 35% in a character × OTU context and included all but six of the extant red algal families (Blinksiaceae, Catenellopsidaceae, Corynocystaceae, Crossocarpaceae, Pseudoanemoniaceae, Rissoellaceae). Figure 1 graphically represents the matrix and clearly highlights four strongly represented loci (EF2, 18S, 28S, *rbc*L). Even though the remaining ten loci were poorly represented, their availability was concentrated in a fixed set of OTUs (Bangiaceae, Compsopogonaceae, Cyanidiaceae, Galdieriaceae, Gigartinaceae, Gracilariaceae, Palmariaceae, Porphyridiaceae, Rhodochaetaceae, Stylonemataceae, Thoreaceae), largely as a consequence of a previous study of these taxa [39].

Our model selection approach showed the importance of partitioning the data to allow differences in substitution processes among data partitions to be captured with composite models of sequence evolution. Of the thirteen potential partitioning strategies that were evaluated, the Bayesian Information Criterion (BIC) selected one that consisted of 8 partitions (plastid ribosomal loci, nuclear ribosomal loci, 1st, 2nd and 3rd codon position of nuclear genes, and 1st, 2nd and 3rd codon position of organelle genes) (Additional file 1). The second order Akaike Information Criterion (AICc), on the other hand, selected a more complex strategy consisting of 13 partitions (23S rDNA, 16S rDNA, 28S rDNA, 18S rDNA, 1st, 2nd and 3rd positions of nuclear genes, 1st, 2nd and 3rd codon positions of plastid genes, and 1st, 2nd and 3rd codon positions of mitochondrial genes) (Additional file 1). As reasoned in the Discussion, we have run our ML searches with the less complex strategy and our Bayesian inferences with the more complex one.

### Phylogenetic results

**Figure 2:**
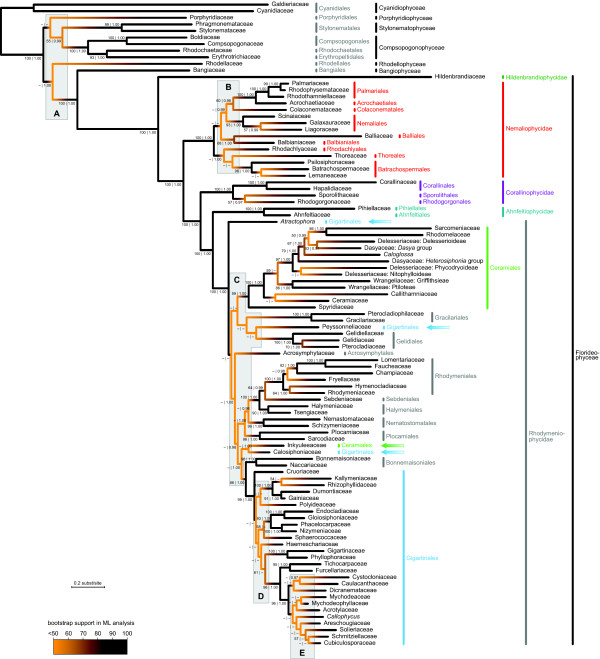
**Red algal tree of life with current taxonomic classification**. The tree was reconstructed using Bayesian phylogenetic inference of DNA data mined from GenBank (Figure 1). Branch colors indicate statistical support of the clades: whereas black branches are strongly supported, the orange parts of the tree are poorly resolved. Intermediate colors represent intermediate support (see gradient legend). Five poorly supported regions are indicated with gray boxes (A-E). Numbers at nodes indicate branch support given as bootstrap values from maximum likelihood analysis before the vertical bar and Bayesian posterior probabilities after the vertical bar. Values are only shown if they exceed 50 and 0.95, respectively.

The phylogenetic tree obtained with Bayesian inference and its correspondence to the current classification of red algae are shown in Figure 2. Although most of the relationships in our tree correspond to results of previous studies, the phylogeny in Figure 2 represents the most complete red algal tree of life published to date. The ML tree is consistent with the Bayesian tree except in some poorly supported regions (Additional file 2). The approximately unbiased (AU) test shows that the BI tree is not significantly less likely than the ML tree (Table 1).

**Table 1:** Likelihood based topological tests

	lnL	BV	**P**_**AU**_
Bayesian tree	-185,594.97	0%	0.403

Ceramiales	-185,607.63	5%	0.186
Gigartinales	-185,635.98	0%	0.574

region A	-185,622.35	0%	< 0.001
region B	-185,678.38	0%	< 0.001
region C	-185,818.04	0%	< 0.001
region D	-185,686.91	0%	0.001
region E	-185,708.05	0%	< 0.001

Various alternative topologies are compared to the ML topology using an AU test. For each alternative topology (rows of the table), the lnL of the alternative topology is given along with the percentage of occurrences of the alternative topology in the unconstrained bootstrap analysis (BV), and the P-value of the AU test on a larger set of trees. On the first data line, the Bayesian tree is compared to the ML tree. In this case, the null hypothesis of the AU test is that the ML tree is not significantly more likely than the BI tree. In the middle part of the table, each of the non-monophyletic orders is listed along with the lnL of the topology in which the order is constrained to be monophyletic. In this case, the null hypothesis of the AU test is that unconstrained and constrained topologies are equally likely. In the bottom part of the table, the possibility that the poorly resolved regions represent hard polytomies is tested. The listed lnL are for the trees in which one of the poorly resolved region was collapsed, and in this case the null hypothesis of the AU test is that uncollapsed and collapsed topologies are equally likely. The lnL of the unconstrained, uncollapsed topology is -185,569.97.

The phylogenetic tree matches the current red algal classification very well, largely because the latter derives from previous molecular studies [39,42,45]. It is noteworthy that all classes, subclasses and most orders are monophyletic in our tree. Only two out of 33 orders were non-monophyletic (Ceramiales and Gigartinales). We used the AU test to evaluate whether trees in which the non-monophyletic orders are forced to be monophyletic have significantly lower likelihoods than the inferred ML tree. The AU test resulted in a 95% confidence set of 33 trees, including the tree in which Ceramiales were monophyletic and the tree in which Gigartinales were monophyletic (Table 1).

Statistical support, measured as bootstrap values, is shown in Figure 2 with a color gradient from black (high support) to orange (low support). In general, the tree is well-supported, especially when compared to previous studies with lower gene sampling. Most importantly, large parts of the backbone of the tree are recovered with maximum statistical support (PP = 1.00, BV = 100). Nonetheless, there are several regions in the tree where support is insufficient to allow firm conclusions. This is most pronounced in the boxed regions in Figure 2, indicated with letters A through E. Although there are other clades with low support in the tree, we will focus on these boxed regions because they represent the most significant gaps in our knowledge about the red algal tree of life. We used the AU test to evaluate the possibility that the regions represent hard polytomies, i.e. polyfurcations stemming from multiple, virtually instantaneous speciation events. This possibility was rejected with high significance for each of the five regions: none of the trees with hard polytomies was contained in the 95% confidence set (Table 1).

### Present data availability

Because resolving the five poorly supported regions will be among the future research priorities, we have summarized the present level of data availability for each of them and estimated the difficulty of resolving them based on a number of simple statistics and with simulation studies.

The most ancient unsupported region (region A), with an estimated late Mesoproterozoic to early Neoproterozoic age [29], has the highest data availability (Table 2, Additional file 3) because it has been targeted previously with broad gene sampling [39]. Even though the old age of this region may pose problems, the intermediate node density may facilitate its resolution. Regions B and C are of intermediate age (likely Neoproterozoic). Current data availability for these regions is meager to poor but their intermediate node densities indicate that these regions may not be very difficult to resolve with confidence. Data availability for the last two regions (D & E) is poor, but data overlap among the few sampled loci is fairly high. Based on their relatively recent age (likely Paleozoic) one may anticipate that these regions are relatively easy to resolve with confidence but this may be hampered by their high node density.

**Table 2:** Data availability, relative age and node density of poorly supported regions

	informative loci	data overlap	relative age	node density
region A	9 → 64.3%	100%	0.88 - 0.97	0.529
region B	4 → 28.6%	83.3%	0.35 - 0.53	0.449
region C	7 → 50.0%	60.3%	0.33 - 0.53	0.548
region D	5 → 35.4%	57.5%	0.34 - 0.43	0.787
region E	3 → 21.4%	75.8%	0.14 - 0.25	1.000

The four statistics presented in this table describe the current data availability for each of the five poorly supported regions and the relative difficulty of resolving them. The proportion of potentially informative loci and the data overlap among potentially informative loci measure current data availability. Potentially informative loci are those that are present for more than three of the OTUs in the matrix. Data overlap is given as the average relative edge weight in the intersection graph of informative loci (see methods). The relative age and node density may indicate how difficult resolving the region will be. The relative age represents how ancient the region is, on a scale from zero (the present) to one (the root of our tree). The node density index is proportional to the number of nodes that need to be resolved per time unit (see methods). The partial data availability matrices for each region can be found in Additional file 3.

### Future data requirements

**Figure 3:**
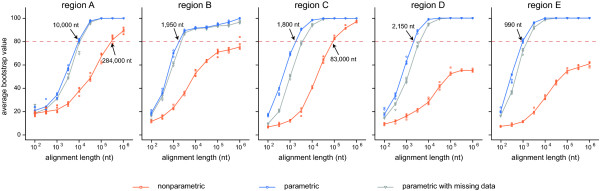
**Estimated data requirement for resolving the five poorly supported regions**. Each graph shows how average bootstrap support increases as a function of alignment length for three types of simulations: nonparametric resampling of the empirical alignment (orange), parametric simulation of data (blue) and parametric simulation followed by introduction of missing data (gray). The approximate amount of data required to resolve a region can be derived for each simulation type by specifying a desired level of bootstrap support (e.g., the dashed line drawn at 80) and deducing the corresponding alignment length on the x-axis. Note that the x-axis uses a logarithmic scale. The lines connect the means of the five values of each condition.

Simulation studies were carried out to estimate the amount of data that will be needed to confidently resolve each poorly supported region. Figure 3 shows how the average bootstrap support of branches in the regional trees increases as a function of alignment length. In order to derive the alignment length required to resolve a region, one must first define the level of bootstrap support the average node should have for the region to be considered resolved. We have added a dashed line at 80% bootstrap support for illustrative purposes. Subsequently, the estimated alignment length required to resolve the region to that level of bootstrap support can be deduced by seeing where the dashed line crosses the line fitted through the data points and reading the corresponding value on the x-axis. It is immediately obvious that the parametric and non-parametric simulation types yielded widely divergent results. Parametrically simulated datasets always resulted in much better resolved trees than nonparametrically generated datasets (blue vs. orange lines). As a consequence, the estimated alignment length required for resolution of a region is small if one attaches more importance to the parametric simulation results (blue line) and much larger if one chooses to use the nonparametric simulation results (orange line). Missing data in the nonparametrically resampled alignments is among the many causes that may be at the base of this discrepancy (see Discussion). In order to estimate the effect of missing data, the parametric simulations were repeated with the same amount of missing data present in the nonparametric datasets. These results (gray line) are intermediate between those of the other two simulation types.

### Spectral partitioning

In order to evaluate whether the signal between natural data subdivisions (gene type, genome and locus) is in conflict, we investigated whether these natural data subdivisions corresponded to spectral partitions. Spectral partitioning subdivides characters in an alignment into a prespecified number of clusters based on character compatibility [46]. Characters in the same cluster are more phylogenetically compatible with each other than they are to characters in different clusters. Our results suggest that there is no strong correspondence between the spectral partitions and the natural data subdivisions (Additional file 4). In contrast, our results show that the amount of conflict between characters within the natural subdivisions exceeds the amount of conflict among natural subdivisions in the majority of cases (exceptions will be mentioned in the discussion). Spectral partitioning into different numbers of clusters yielded similar results and only the results of the analysis with three spectral partitions are shown in Additional file 4.

## Discussion

We have reconstructed a red algal tree of life at the family level based primarily on data mined from GenBank. Our principal goal in reconstructing this tree was to identify the well-resolved parts and the remaining uncertainties in the tree, the latter engendering a better knowledge about the gaps in currently available data and leading to clearly defined research priorities for future efforts to resolve the red algal tree of life.

### Improved red algal Tree of Life

As one would anticipate, the tree we obtained was more complete and better resolved than those of most previous studies with lower gene and taxon sampling. This is likely to be due to two factors. First, a considerably larger amount of data is used in this study, both in terms of taxon and gene sampling. Second, we have carefully selected models of sequence evolution that can capture various complexities of the sequence data by allowing different model parameter values for different data partitions. It has been well established that appropriately partitioned models of sequence evolution yield a better fit to empirical datasets than simple models [47-49] and simulation experiments have shown that phylogenetic analysis with suitably partitioned models results in more accurate trees [50]. For our dataset, the Akaike selection criterion recommended finer subdivision of the data (13 partitions) than the Bayesian information criterion (8 partitions). We have chosen to use the more complex model for Bayesian phylogenetic inference and the less complex model for ML searches. Although somewhat arbitrary, this choice is endorsed by theoretical studies showing that whereas BI is sensitive to underparametrization [51], ML optimization is more liable to suffer from overparametrization [52,53]. Specifically, Bayesian analyses using overly simple models tend to yield overly high posterior probabilities [51], which is undesirable considering that we aim for a realistic assessment of uncertainties in our red algal tree of life.

### Causes of remaining uncertainties

Despite the fact that our phylogeny is better resolved than many previous trees, it clearly shows that a lot of work remains to be done to resolve the red algal tree of life. Using a simple visualization technique that maps bootstrap support on the tree as colors along a color gradient, five poorly supported regions of the tree could be readily identified (Figure 2). Poor resolution in phylogenetic trees can have several potential causes. The first possibility is that several speciation events have occurred virtually simultaneously. In this case, the biologically correct phylogeny contains hard polytomies. This does not seem to be the case for the poorly supported regions in our red algal tree because our AU test strongly rejects the topologies in which the poorly supported regions were collapsed. In this context it is important to note that each one of our tests focuses on an entire region being a hard polytomy. So the test only rejects the possibility that the entire region is a hard polytomy, but it is still possible that smaller hard polytomies exist within a region.

If not a result of biological reality, the poorly resolved regions must follow from inadequacy of the dataset or failure of the phylogenetic methods. Many studies have shown that inappropriate inference methods can fail to recover the correct phylogenetic tree from DNA sequences [54-57]. We have taken some precautions to avoid problems of this nature. First, we have used inference methods that make explicit use of models of sequence evolution because these are known to outperform alternative techniques under a wide range of conditions [56]. Second, we have accounted for various complexities in our composite dataset by carrying out extensive model selection procedures and performing phylogenetic analyses with models that show a good fit to the data.

The last possible cause for the poorly supported regions option is that the dataset is inadequate for resolving them. Two main factors can contribute to failure of a dataset to resolve a phylogeny: conflict in the data and lack of information in the data. Conflicting signals most commonly occur between genome partitions or between individual genes. However, this does not appear to be the case in our dataset because spectral partitions based on site compatibility do not correspond to natural partitions. This lack of correspondence indicates that conflict between natural data subdivisions (gene type, genome and locus) is smaller than the conflict between sites within each of the natural subdivisions. The information content of a phylogenetic data matrix depends on the number of characters, the number of taxa and the phylogenetic informativeness of each site [13,58-60]. Because the taxon sampling of our study is nearly complete at the family level, the number of characters and the amount of missing data in our DNA matrix (Figure 1) are more likely to be at the base of the poor resolution. To examine this in more detail and estimate how much data would be necessary to resolve the poorly supported regions, we have calculated several statistics and carried out simulation studies.

### Future data requirements

The current data availability statistics, along with the relative age and the node density of each poorly supported region permit more insight into the possible causes of the lack of resolution and, along with the results from the simulation experiments, allow us to make more specific recommendations. For this discussion, we will consider an average bootstrap value of 80% (dashed line in Figure 3) acceptable support.

Region A consists of the relationships between a few classes near the base of the red algal tree. Despite having the highest proportion of potentially informative loci of all regions and maximal data overlap, the relationships among these classes have not been resolved confidently [39]. This is probably due to a combination of the intermediate node density in this region and its age. Resolution of the ancient relationships among the lineages in this region of the tree will require the generation of large amounts of additional data. Parametric simulations require almost 10,000 sites to reach acceptable support and nonparametric resampling suggests that almost 284,000 sites will be needed.

Region B encompasses the order-level relationships of the Nemaliophycidae. Maximum likelihood and Bayesian trees differed in some of these relationships and some of the nodes in Figure 2 that were poorly supported in ML bootstrap analyses did receive high posterior probabilities in the Bayesian analysis. As mentioned above, different partitioning strategies were used for our ML and Bayesian analyses, which may have caused the discrepancy. The number of potentially informative loci is remarkably low for this region. Given the relatively low node density and intermediate age of this region, one would expect that this region would not be too difficult to resolve. Parametric simulations confirm this: they suggest that ca. 1,950 sites should suffice to achieve acceptable support. This is in stark contrast with the nonparametric resampling method, which never reached the 80% threshold. The spectral partitioning results offer some initial insights into why the nonparametric results are so pessimistic. The 16S and 23S genes have a markedly different spectral composition than the other loci, and the contrast is especially strong if 16S and 23S are compared to 18S (Additional file 4). Remarkably, this effect is no longer apparent when comparing the spectral composition of genomes: nuclear and plastid genomes show a similar spectral composition.

Region C consists of the apparently sudden radiation of lineages at the base of the Rhodymeniophycidae. Even though the statistics in Table 2 indicate an intermediate node density, the fact that most nodes are situated close to the beginning of the epoch spanned by this region and only a few are near the end of the epoch gives us reason to believe that region C represents a rapid radiation. This region also features the most pronounced differences between the maximum likelihood and Bayesian trees. As was the case for region B, Bayesian support values are high (PP > 0.95) for a handful of nodes in region C that were not present or very weak in the set of ML bootstrap trees. Other studies have also indicated the sensitivity of relationships in this region to methodology, gene and taxon sampling [40,43,44]. A further discussion of these results is beyond the scope of this paper - for now, it suffices to conclude that there is considerable uncertainty about the relationships in region C, which should form a future research priority. The combination of the large number of lineages emanating in this region, its old age (probably Neoproterozoic) and the substantial previous effort that has not led to a solid understanding of its evolution may suggest that this region will be a tough one to resolve. Nonetheless, parametric simulations required only ca. 1,800 sites to achieve acceptable support. Nonparametric resampling reached the 80% threshold at ca. 83,000 nucleotides.

Region D encompasses the relationships among some subgroups of the Gigartinales. Data availability and data overlap are currently insufficient to resolve this region, probably due to the relatively high node density. The parametric simulation results confirm the difficult nature of this region: 2,150 sites were required to resolve it to an average bootstrap support of 80%. This requirement is higher than that of regions B and C, which are both considerably older. Nonparametric simulations did not reach the 80% threshold.

Region E represents a relatively recent radiation of gigartinalean families. The combination of low data availability and high node density is probably responsible for the lack of resolution in this region. Our spectral partitioning results also suggest that conflict may be present between the signal contained in the 18S rDNA alignment and that of the 28S rDNA dataset (Additional file 4). Even though parametric simulations suggest that this is a relatively easy region to resolve (ca. 990 nucleotides), our nonparametric resampling did not reach acceptable levels of support.

From these summaries it can be concluded that the five poorly supported regions stem from a diversity of causes and that resolving them will likely require different kinds of datasets. It is also clear that the amount of data that will be needed to resolve each of the regions is still difficult to estimate due to the large differences between the parametric and nonparametric simulation results. This will be discussed in more detail in the next section. Irrespective of the exact data requirements, it is clear that more data are needed to resolve the red algal tree of life and that a dual approach will be best suited to address the variety of phylogenetic questions in the five unresolved regions. First, high-throughput genomics efforts will be needed to resolve region A and perhaps region B. Such efforts could consist of organelle genome sequencing, EST data generation or a combination of both. Second, the relationships in regions C, D and E require generating large multi-locus datasets for a broad selection of Rhodymeniophycideae for which targeted PCR amplification may be preferable to high-throughput genomics because of the large number of taxa involved and lower estimated data requirements. In addition to resolving the poorly supported regions in our tree, generating data for the six families absent from our tree should be a research priority.

### Limitations of simulation approach

Several studies have used simulation experiments to estimate the amount of data needed to resolve phylogenetic questions. Both parametric [26,61] and nonparametric [22,62,63] approaches have been used widely. Our results clearly demonstrate some limitations of this approach. Without exception, the nonparametric simulations suggested a markedly more pessimistic image of data requirement than the parametric simulations. Several elements of the experimental design are likely to have an appreciable contribution to the difference between our parametric and nonparametric simulation results.

First, the simple model of sequence evolution used in the simulations yields alignments that are not as complex as empirical data matrices. As a consequence, parametrically simulated datasets produce higher support values because the ML inference uses the true model. More biological realism can be added to parametric simulations by incorporating gene tree heterogeneity [61] or using highly complex models of sequence evolution [64].

Second, the nonparametric approach used here has the disadvantage that no genuinely new data are added to the data matrix when it is resampled beyond the original alignment length. This will lead to a more pronounced effect of signal present in the dataset but nodes for which there is little signal or for which there are equal amounts of conflicting signal can be expected to remain unresolved when no effectively new data are added. This effect thus depends on the amount of data present in the original alignment. In our case, the length of the regional alignments decreases in this order: A > C > B > D > E. Thus, all regions for which the 80% support threshold is not reached (B, D, E) start out with relatively small alignments. Resolving the effect of this issue requires extra theoretical work.

Third, missing data present in the empirical data that are resampled in the nonparametric simulations can be expected to reduce bootstrap support to some extent. It is important to note that our regional alignments have much less missing data than our global data matrix because only the potentially informative loci are included in them. Our parametric simulations with the same distribution of missing data than the original regional alignments show that missing data in our regional alignments has an effect, yet it explains only a small fraction of the difference between parametric and nonparametric simulations (Figure 3).

In conclusion, it is evident that the alignment lengths suggested by parametric simulation are too optimistic and those of nonparametric simulations too pessimistic. For that reason, we have interpreted their respective predictions as lower and upper bounds on future data requirements. The predictions of the parametric simulations have the advantage that they can be more directly compared between regions to evaluate the relative difficulty of resolving them with a certain degree of bootstrap support.

### Complementary strategies

In addition to generating supplementary data, further improvements of the experimental design and analysis techniques could also contribute to the robustness of results. First, the assumption of character independence can be relaxed by using special models of sequence evolution inspired by specific characteristics of the studied molecule such as RNA secondary organization, codon structure and across-site process heterogeneity [65-69]. Second, restricting analyses to subsets of the tree requires less data exclusion because there is less alignment ambiguity and may allow more accurate estimation of model parameters relevant to that region of the tree. Mishler's compartmentalization approach could be useful in this context because it allows combining phylogenetic insights at various levels in a global phylogeny [70,71]. Third, resolving ancient phylogenetic relationships can benefit from techniques that improve the signal-to-noise ratio in phylogenetic datasets, for example by selective removal of fast-evolving sites [72]. Given that the red algae are more than a billion years old, all five unresolved regions could be classified as ancient. Finally, it is worth noting that certain aspects of experimental design can also affect tree inference. Taxon sampling is especially relevant here. In this context, our analysis may suffer to some extent from the use of families as OTUs. This approach leads to relatively long external branches, which may result in lower internal support values. Increasing the taxon sampling within each family can easily solve this.

### Taxonomic perspectives

Finally, the resolution of the red algal tree of life will engender a better, more natural classification of the red algae. Even though the present classification closely matches our molecular phylogeny, two currently recognized orders were non-monophyletic in our tree. It must be noted, however, that the component lineages of these orders are situated in the poorly supported regions and that monophyly of the orders is not rejected with statistical confidence (Table 1). Nonetheless, the non-monophyly of the orders in question could be anticipated from previous work. The inclusion of the Inkyuleeaceae in the Ceramiales has been questioned in several studies [73,74]. The non-monophyly of the Gigartinales is also not surprising. Years of controversy regarding the distribution of families between this order and the Cryptonemiales resulted in a surrender tactic in which Kraft and Robins [75] simply merged the two orders considering this the best step forward for a total re-evaluation of this complex. Since that time multiple discordant elements have been moved out to other or new orders in an effort to render a monophyletic Gigartinales, a task that continues to this day [43,76].

## Conclusions

Fifteen years of molecular phylogenetic research have changed radically our perspectives on red algal relationships at all taxonomic levels. These foundation studies have either had limited taxonomic objectives or were based on one or only a few genetic regions. The present data mining effort was initiated to take this area of study to the next level, one encompassing analyses of a supermatrix containing many loci and nearly all red algal families. In doing this we have confirmed many of the earlier findings, but have more critically highlighted five regions of low resolution and provided insights as to future directions to resolve these conundrums. More specifically, we have shown that the currently unresolved regions stem from a diversity of causes and that resolving them will require different approaches. We propose a dual approach consisting of high-throughput genomic data to resolve the two most difficult phylogenetic problems (regions A and B) and the development of targeted multi-locus datasets of to resolve the remaining problems in the Rhodymeniophycidae (regions C-E). The present study illustrates how data mining approaches can guide the design of projects aimed at reconstructing the tree of life and will hopefully provide our colleagues and us with the necessary groundwork to move this objective forward.

## Methods

### Dataset composition

All available red algal DNA sequences were acquired from GenBank release 160 and stored in a local database. EST data and sequences longer than 5000 bases were excluded. Ribosomal RNA and protein-coding genes from complete organelle genomes were added back as separate entries. Sequences belonging to the fourteen target loci (Figure 1) were extracted and stored in separate databases (one for each locus). The sequence extraction process consisted of three steps. A first set of sequences belonging to the loci of interest was extracted based on a database of accession numbers that was generated in the framework of a literature survey and meta-analysis [45]. Second, annotations and keywords in the description of these entries were subsequently used to extract a second set of entries from the local database. The assignment of these sequences to the loci was double-checked with BLAST scores. A third set of entries was extracted by performing BLAST searches of sequences annotated in the previous steps against the remainder of red algal sequences in the local GenBank database for each target locus separately. Sequences yielding high BLAST scores were added to the appropriate files after manual screening of the annotations. Additional sequence data were generated following previously published protocols and added to the databases [44,77,78]. Newly generated sequences are indicated with an asterisk in the data matrix (Additional file 5).

After introns had been removed from the sequences, they were given a quality score corresponding to their length minus the number of ambiguous base calls. The highest-scoring sequences of each red algal family were selected. For a few families of doubtful status, we refined the classification and used intrafamilial groupings as OTUs. The taxonomic database used for this purpose was based on a recent classification scheme [79], with some minor modifications to add extra taxonomic levels within certain families and reflect recent work [74,80-82]. The highest-scoring sequences (see Additional file 5) were stored in fasta files and aligned by eye. Gap-rich and ambiguous regions were discarded. The fourteen resulting alignments were concatenated into a single supermatrix. Alignments of individual loci and the supermatrix will be made available through TreeBase [4] and at http://www.phycoweb.net.

### Model selection

A suitable partitioning strategy and partition-specific substitution models were selected in a multi-step process illustrated in Additional file 1. Initially, base frequencies of different genes and codon positions were visualized to obtain a gross idea of base frequency differences among potential data partitions. This preliminary information and knowledge about the genomic compartment of the loci led us to identify thirteen partitioning strategies for further consideration (more details in Results). Subsequently, a suitable partitioning strategy and partition-specific models of sequence evolution were selected using the Bayesian Information Criterion (BIC). This selection procedure consisted of three steps. For the purpose of model selection, a guide tree was obtained by carrying out a second-level ML search on the unpartitioned dataset with a HKY + Γ_8 _model with TreeFinder [83]. The first step of the procedure was to optimize the likelihood of the dataset for thirteen partitioning strategies, assuming the guide tree and separate HKY + Γ_8 _models for each partition. The six best-scoring partitioning strategies were retained for further analysis. In the second step, models of sequence evolution were selected for individual partitions using the BIC. For each partition present in the six retained partitioning strategies, six different nucleotide substitution models were evaluated (F81, F81 + Γ_8_, HKY, HKY + Γ_8_, GTR, GTR + Γ_8_). The likelihood of observing the data of each partition was optimized under these models, assuming the guide tree pruned to the taxa present in the partition. In the third step, the six partitioning strategies retained in the first step were re-tested, this time applying the best scoring model of sequence evolution identified in the second step to the partitions. Both the BIC and the second order Akaike Information Criterion (AICc) were evaluated during this step. All likelihood optimizations and information criterion computations were carried out with TreeFinder.

### Bayesian phylogenetic inference

The phylogenetic relationships among taxa were inferred using Bayesian inference (BI) and maximum likelihood searches (ML). Bayesian phylogenetic inference was carried out with MrBayes v.3.1.2 [84]. The analysis used the composite model selected with the AICc, with all parameters unlinked among partitions. Partition rates were allowed to vary under a flat Dirichlet prior. Five runs of four incrementally heated chains were run in parallel (temperature increment = 0.5). The chains were run for 35 million generations, with a sample frequency of 1000. MrBayes' default priors, proposal probabilities and other settings were used. Convergence of the runs was assessed by visual examination of parameter traces and marginal densities using Tracer v.1.4 [85]. An appropriate burn-in value was determined using the automated method proposed by Beiko et al. [86]. Their method was applied to each run individually, with a sliding window of 1000 samples, yielding five different burn-in values. Because two out of the five runs converged onto suboptimal likelihoods and a third run yielded low effective sample sizes (ESS) for a subset of parameters despite convergence of the likelihood, the posterior distribution of trees was summarized from the MCMC output of the remaining two runs using the highest burn-in value obtained across the two runs in question.

### Maximum likelihood searches

Maximum likelihood analyses were carried out with TreeFinder. This software allows tree searches under complex (partitioned) models within reasonable time by implementing fast tree search heuristics, with the trade-off that searches can get stuck on local likelihood optima. To achieve a more expansive coverage of tree space, tree searches were started from a multitude of start trees. The search procedure consisted of three rounds of ML searches from different start trees. First, 100 start trees were generated by randomly modifying the guide tree used for model selection by a number of nearest neighbor interchange (NNI) steps. The amount of change from the guide tree was 200 and 500 NNI steps (50 replicates each). ML tree searches were carried out from each of these start trees. Out of the set of resulting ML trees, the three with the highest likelihood were retained for a second round of NNI modifications (100 NNI steps, 30 replicates). ML searches starting from the new set of start trees were carried out and the three highest-scoring trees were used for a last round of NNI modifications (20 and 50 NNI steps, 20 replicates each). The tree with the highest likelihood resulting from the last round of analyses was selected as the ML tree. All analyses used the composite model selected with the BIC, but parameter estimates were re-optimized during the ML search. The second-level tree search was used and partition rates were optimized under the proportional model. Branch support was calculated by non-parametric bootstrapping (1000 replicates). Bootstrap replicates were started from the ML tree.

### Topological hypothesis testing

The presence of a few non-monophyletic orders in our phylogenetic tree prompted us to evaluate the statistical significance of this non-monophyly. Similarly, we wanted to evaluate the statistical significance of differences between the ML and BI tree and of trees in which poorly resolved regions were collapsed into a hard polytomy. We used the approximately unbiased (AU) test, which is based on nonparametric resampling using the likelihood criterion, to identify a 95% confidence set of trees from a larger set of trees. The large set of trees we used in this analysis included the ML tree, the eight alternative topologies from Table 1, and the ML trees of 1000 bootstrap searches.

The alternative topologies were inferred as follows. The Bayesian tree was taken from the BI described above. For each of the non-monophyletic orders, we inferred a ML tree in which the order was constrained to be monophyletic. For each of the poorly resolved regions (see below), we constructed a tree in which the region in question was collapsed and subjected this tree to likelihood optimization. For all trees, site-specific likelihoods were calculated with TreeFinder [83]. Subsequently, the AU test was performed with CONSEL v.0.1i [87], using default settings. We verified whether each of the eight alternative topologies was present in the 95% confidence set.

### Characterization of poorly supported regions

In order to identify future research priorities, we aimed to (1) identify poorly supported regions of the phylogenetic tree, (2) summarize the current data availability for the taxa in question and (3) estimate how hard it may be to resolve the poorly supported regions.

Branch support (ML bootstrap values) was visualized with TreeGradients v.1.04, allowing straightforward visual identification of poorly supported regions [88]. By plotting ML bootstrap values on the Bayesian phylogenetic tree, regions featuring poor support can result either from genuinely low bootstrap support or from disagreement between Bayesian and ML results, both of which are undesirable.

First, we tested the possibility that the poorly supported regions represent hard polytomies. For each poorly supported region, we constructed a tree in which the region in question was collapsed. These trees were included in an AU test to verify whether they are included in the 95% confidence set of trees (see previous section). If a collapsed tree is not included in the 95% confidence interval, its likelihood is significantly lower than that of the uncollapsed tree, which can be taken as an indication that the unsupported region does not represent a hard polytomy [22].

For further, more detailed analyses, the well-supported lineages emanating from them were identified and designated as OTUs. We constructed a partial data availability matrix for each poorly supported region. This involved the generation of consensus sequences for OTUs consisting of more than one red algal family. From this matrix, we calculated the fraction of potentially informative loci currently available for analyzing the relationships among the OTUs of interest. A potentially informative locus is defined as a locus that is present for at least four OTUs of interest. The fraction of potentially informative loci is simply calculated as the number of potentially informative loci divided by the total number of loci considered in this study (14). The number of potentially informative loci alone is not always a good indicator of data availability because there also has to be sufficient taxon overlap between loci to yield resolved trees. For that reason, we calculated a statistic representing the amount of taxon overlap between the potentially informative loci. This was done by creating an intersection graph of the potentially informative loci [89]. The edges connecting different loci were weighted by the number of taxa shared between them, divided by the total number of taxa. The statistic we will report as a measure of data overlap is the mean edge weight of the intersection graph. It is important to note that only potentially informative loci were used to construct the graphs and calculate the statistics.

In an attempt to further quantify how difficult it may be to resolve the poorly supported regions, two additional statistics were calculated. First, the relative age of the regions was inferred by fitting a relaxed molecular clock model. We fit a lognormal model of rate evolution with PhyloBayes [90], based on the Bayesian phylogenetic tree, a dataset consisting of the four most densely sampled loci (EF2, 18S rDNA, 28S rDNA &*rbc*L), and giving the root node an arbitrary age of 1. Second, we calculated the node density for each region. Our index of node density consisted of the number of nodes that would need to be present in the region for it to be fully bifurcating, divided by the time span of the region and rescaled so that the region with the highest node density had a value of 1. Our node density index is proportional to the rate of cladogenesis in the region, with high values indicating fast cladogenesis, making the region in question more difficult to resolve.

### Future data requirements

We carried out a set of simulation studies to estimate how much data will be needed to resolve the poorly supported regions. Our approach consisted of both nonparametric and parametric bootstrapping using alignments of different lengths and evaluation of the resolution of resulting trees as a function of alignment length. The following analyses were carried out for each region separately.

First, a subalignment and a subtree of the region were generated by treating the well-supported lineages emanating from the poorly supported region as OTUs. If lineages emanating from the region comprised multiple taxa, the entire clade was replaced with a single branch. The length of this branch was set to be the average path length between the ancestral node and each of the descendent leaf nodes. All subtrees were strictly bifurcating but typically included some very short internal branches. In the regional alignments, OTUs containing multiple taxa were represented by majority-rule consensus sequences. Regional alignments were reduced to the set of potentially informative loci. One outgroup sequence was included with each regional alignment. This sequence belonged to the sister group of the poorly supported region. If the sister group contained multiple taxa, a consensus sequence was used as explained above.

For both the nonparametric and parametric approaches, sequence alignments of different lengths between 10^2 ^and 10^6 ^nucleotides were generated, with 100 replicate alignments per alignment length. For the nonparametric approach, the regional alignment was resampled with replacement until the desired alignment length was reached. For the parametric approach, alignments of the desired length were generated by simulating sequence evolution along the regional subtrees under a GTR + I + Γ_4 _model with Seq-Gen v.1.3.2 [91]. The parameters used for the simulation were obtained by optimizing a GTR + I + Γ_4 _model for the complete alignment and ML tree with RAxML [92]. A third set of simulations aims to introduce extra realism in the parametric simulations by introducing missing data. Missing data was introduced in the same amount and distribution among sites and taxa as in the empirical alignments.

All alignments were subjected to ML phylogenetic inference in RAxML, using a GTR + I + Γ_4 _model. We summarized the 100 resulting ML trees per condition by constructing a strictly bifurcating majority rule consensus tree (i.e. without a lower limit on clade presence). The average bootstrap value on the majority rule consensus tree was plotted as a function of alignment length to evaluate how data availability affects tree resolution. All simulations were repeated five times, thus yielding five average bootstrap values per condition to have an idea of the spread of the results. The entire simulation experiment amounted to ML analysis of 67,500 random alignments (5 regions · 3 simulation types · 9 alignment lengths · 100 replicates · 5 repetitions) and was carried out on Ghent University's central HPC facility.

### Spectral partitioning

We examined potential conflicts in phylogenetic signal between natural data subdivisions by comparing them to spectral partitions. The natural subdivisions we used were gene type (coding for protein or ribosomal RNA), genome (nuclear, mitochondrial or plastid) and locus (16S, 18S, 23S, 28S, EF2, *cox*1, *psa*A, *psa*B, *psb*A, *psb*C, *psb*D, *rbc*L, *rbc*S &*tuf*A). Spectral partitioning is a technique that partitions alignments based on character compatibility. More specifically, it clusters the characters with the highest average pairwise compatibility, so that characters in each cluster are more compatible with each other than they are with characters in the other clusters [46]. If the relative contribution of spectral partitions differs strongly between gene types, genomes or loci, this can be taken as evidence for conflict between them. If, on the other hand, similar proportions are found, the conflict within the natural data partitions exceeds the conflict between them, indicating that the different natural partitions contain similar phylogenetic signal.

We applied spectral partitioning to each of the regional subalignments separately. Analyses were run on a web server [93] using the fractional compatibility scoring procedure. Each subalignment was analyzed four times to allow spectral partitioning into two, three, four and five clusters. The contribution of the different spectral partitions to each of the natural data subdivisions was assessed by plotting the fraction of sites belonging to the different spectral partitions for each of the natural data subdivisions. Phylogenetically uninformative sites were not included in these calculations.

## Authors' contributions

HV and ODC conceived of the study. GWS and LLG generated sequence data. HV assembled data and performed phylogenetic analyses. HV, CAM and GWS wrote the manuscript. All authors read and approved the final manuscript.

## Supplementary Material

Additional file 1 **Model selection procedure**. Illustration of the model selection procedure, including results. Click here for file

Additional file 2 **Maximum likelihood phylogeny**. Tree inferred from the 14-locus data matrix using ML inference, with ML bootstrap values at internal nodes. Click here for file

Additional file 3 **Partial data availability matrices for five poorly supported regions**. Four statistics describing current data availability and the relative difficulty of resolving the region are given below the matrices (see also Table 2 in main paper). The proportion of potentially informative loci and the data overlap among potentially informative loci measure current data availability. Potentially informative loci are those that are present for more than three of the OTUs in the matrix. Data overlap is given as the average relative edge weight in the intersection graph of informative loci (see methods). The relative age and node density may indicate how difficult resolving the region will be. The relative age represents how ancient the region is, on a scale from zero (the present) to one (the root of our tree). The node density index is proportional to the number of nodes that need to be resolved per time unit (see methods). Click here for file

Additional file 4 **Spectral partitioning**. The five regional subalignments were subjected to spectral partitioning, a technique that partitions alignments based on character compatibility, the sites most compatible with each other ending up in the same partition. In order to identify potential data conflict between gene types (protein and rDNA), genomes (mitochondrial, nuclear and plastid) and individual loci, we plotted the relative contribution of each spectral partition for each gene type, genome and locus. If the relative contribution of spectral partitions differs strongly between gene types, genomes or loci, this can be taken as evidence for conflict between them. If, on the other hand, similar proportions are found, the conflict within them exceeds the conflict between them, indicating that the different gene types, genomes and loci contain similar phylogenetic signal. Note that the spectral partitions are calculated for each region separately and spectral partitions should thus not be compared between regions as any given site may have been assigned to different partitions for different regions. Click here for file

Additional file 5 **Data matrix with GenBank accession numbers**. List of sequences included in our alignment, with Genbank accession numbers and the species from which they originated. Click here for file
