# Supplementary material for: Data mining approach identifies research priorities and data requirements for resolving the red algal tree of life
Source: BMC Evol Biol. 2010 Jan 20;10:16. doi: 10.1186/1471-2148-10-16 (PMC2826327; doi:10.1186/1471-2148-10-16)
Supplement: Additional file 1 — Model selection procedure. Illustration of the model selection procedure, including results. [file 1471-2148-10-16-S1.PDF]

Additional file 1. Illustration of model selection procedure, including results.

A. Partitioning strategies

Strategy 1
1.1 all data

Strategy 2
2.1 ribosomal loci (23S, 28S, 16S, 18S)
2.2 protein-coding loci (EF2, *cox*1, *psa*A, *psa*B, *psb*A, *psb*C, *psb*D, *rbc*L, *rbc*S, *tuf*A)

Strategy 3
3.1 plastid ribosomal loci (23S, 16S)
3.2 nuclear ribosomal loci (28S, 18S)
3.3 protein-coding loci (EF2, *cox*1, *psa*A, *psa*B, *psb*A, *psb*C, *psb*D, *rbc*L, *rbc*S, *tuf*A)

Strategy 4
4.1 23S plastid ribosomal DNA
4.2 16S plastid ribosomal DNA
4.3 28S nuclear ribosomal DNA
4.4 18S nuclear ribosomal DNA
4.5 protein-coding loci (EF2, *cox*1, *psa*A, *psa*B, *psb*A, *psb*C, *psb*D, *rbc*L, *rbc*S, *tuf*A)

Strategy 5
5.1 ribosomal loci (23S, 28S, 16S, 18S)
5.2 1st codon position of all genes
5.3 2nd codon position of all genes
5.4 3rd codon position of all genes

Strategy 6
6.1 plastid ribosomal loci (23S, 16S)
6.2 nuclear ribosomal loci (28S, 18S)
6.3 1st codon position of all genes
6.4 2nd codon position of all genes
6.5 3rd codon position of all genes

Strategy 7
7.1 23S plastid ribosomal DNA
7.2 16S plastid ribosomal DNA
7.3 28S nuclear ribosomal DNA
7.4 18S nuclear ribosomal DNA
7.5 1st codon positions of all genes
7.6 2nd codon positions of all genes
7.7 3rd codon positions of all genes

Strategy 8
8.1 ribosomal loci (23S, 28S, 16S, 18S)
8.2 1st codon position of nuclear genes (EF2)
8.3 2nd codon position of nuclear genes
8.4 3rd codon position of nuclear genes
8.5 1st codon position of organelle genes (*cox*1, *psa*A, *psa*B, *psb*A, *psb*C, *psb*D, *rbc*L, *rbc*S, *tuf*A)
8.6 2nd codon position of organelle genes
8.7 3rd codon position of organelle genes

Strategy 9
9.1 plastid ribosomal loci (23S, 16S)
9.2 nuclear ribosomal loci (28S, 18S)
9.3 1st codon position of nuclear genes
9.4 2nd codon position of nuclear genes
9.5 3rd codon position of nuclear genes
9.6 1st codon position of organelle genes
9.7 2nd codon position of organelle genes
9.8 3rd codon position of organelle genes

Strategy 10
10.1 23S plastid ribosomal DNA
10.2 16S plastid ribosomal DNA
10.3 28S nuclear ribosomal DNA
10.4 18S nuclear ribosomal DNA
10.5 1st codon position of nuclear genes
10.6 2nd codon position of nuclear genes
10.7 3rd codon position of nuclear genes
10.8 1st codon pos of organelle genes
10.9 2nd codon pos of organelle genes
10.10 3rd codon pos of organelle genes

Strategy 11
11.1 ribosomal loci (23S, 28S, 16S, 18S)
11.2 1st codon position of nuclear genes
11.3 2nd codon position of nuclear genes
11.4 3rd codon position of nuclear genes
11.5 1st codon pos of plastid genes (*psa*A, *psa*B, *psb*A, *psb*C, *psb*D, *rbc*L, *rbc*S, *tuf*A)
11.6 2nd codon pos of plastid genes
11.7 3rd codon pos of plastid genes
11.8 1st codon pos of mitochondrial genes (*cox*1)
11.9 2nd codon pos of mitoch genes
11.10 3rd codon pos of mitoch genes

Strategy 12
12.1 plastid ribosomal loci (23S, 16S)
12.2 nuclear ribosomal loci (28S, 18S)
12.3 1st codon position of nuclear genes
12.4 2nd codon position of nuclear genes
12.5 3rd codon position of nuclear genes
12.6 1st codon pos of plastid genes
12.7 2nd codon pos of plastid genes
12.8 3rd codon pos of plastid genes
12.9 1st codon pos of mitoch genes
12.10 2nd codon pos of mitoch genes
12.11 3rd codon pos of mitoch genes

Strategy 13
13.1 23S plastid ribosomal DNA
13.2 16S plastid ribosomal DNA
13.3 28S nuclear ribosomal DNA
13.4 18S nuclear ribosomal DNA
13.5 1st codon position of nuclear genes
13.6 2nd codon position of nuclear genes
13.7 3rd codon position of nuclear genes
13.8 1st codon pos of plastid genes
13.9 2nd codon pos of plastid genes
13.10 3rd codon pos of plastid genes
13.11 1st codon pos of mitoch genes
13.12 2nd codon pos of mitoch genes
13.13 3rd codon pos of mitoch genes

B. Selection approach for partitioning strategy and models of sequence evolution

step 1 – identify set of suitable partitioning strategies

Strat.	BIC	ΔBIC
1	401411	24174
2	396783	19545
3	396579	19341
4	396574	19336
5	386058	8821
6	385855	8617
7	385850	8612
**8**	**377462**	**225**
**9**	**377249**	**11**
**10**	**377238**	**0**
**11**	**377471**	**233**
**12**	**377257**	**19**
**13**	**377245**	**7**

six best partitioning strategies retained for further analysis

step 2 – identify suitable partition-specific models of sequence evolution

Partition	F81	F81 + Γ_8	HKY	HKY + Γ_8	GTR	GTR + Γ_8
8.1 = 11.1	129440	115387	125319	110738	124886	**110395**
9.2 = 12.2	85948	75451	83788	72966	83556	**72798**
9.1 = 12.1	43694	40006	41589	37611	41195	**37286**
10.1 = 13.1	21441	20382	20619	19458	20395	**19254**
10.2 = 13.2	22303	19676	21023	18217	20872	**18106**
10.3 = 13.3	54833	47615	53358	45862	53138	**45707**
10.4 = 13.4	31219	28204	30545	27460	30520	**27448**
8.2 = 9.3 = 10.5 = 11.2 = 12.3 = 13.5	18737	16750	18505	16417	18191	**16157**
8.3 = 9.4 = 10.6 = 11.3 = 12.4 = 13.6	10875	9666	10882	9672	10561	**9447**
8.4 = 9.5 = 10.7 = 11.4 = 12.5 = 13.7	57014	55522	54578	51970	53644	**51727**
8.5 = 9.6 = 10.8	55395	49651	54211	48016	52356	**46709**
8.6 = 9.7 = 10.9	26337	24419	26336	24418	25981	**24096**
8.7 = 9.8 = 10.10	128656	124994	124843	117382	122046	**116974**
11.5 = 12.6 = 13.8	49780	44622	48838	43314	47180	**42107**
11.6 = 12.7 = 13.9	24559	22772	24561	22775	24260	**22494**
11.7 = 12.8 = 13.10	113689	110739	110121	104046	107565	**103694**
11.8 = 12.9 = 13.11	5809	5222	5550	4888	5382	**4818**
11.9 = 12.10 = 13.12	2004	1875	2003	1870	1956	**1835**
11.10 = 12.11 = 13.13	14971	14373	14688	13525	14396	**13477**

step 3 – re-evaluate six best partitioning strategies with selected partition-specific models of sequence evolution

Strat	BIC	ΔBIC	AICc	ΔAICc
8	374491	340	372430	668
9	**374151**	**0**	372012	250
10	374179	28	371882	120
11	374607	456	372311	549
12	374311	160	371937	175
13	374293	142	**371762**	**0**
