# Supplementary material for: Data mining approach identifies research priorities and data requirements for resolving the red algal tree of life
Source: BMC Evol Biol. 2010 Jan 20;10:16. doi: 10.1186/1471-2148-10-16 (PMC2826327; doi:10.1186/1471-2148-10-16)

Additional file 2. Maximum likelihood phylogeny. Tree inferred from the 14-locus data matrix using ML inference, with ML bootstrap values at internal nodes.

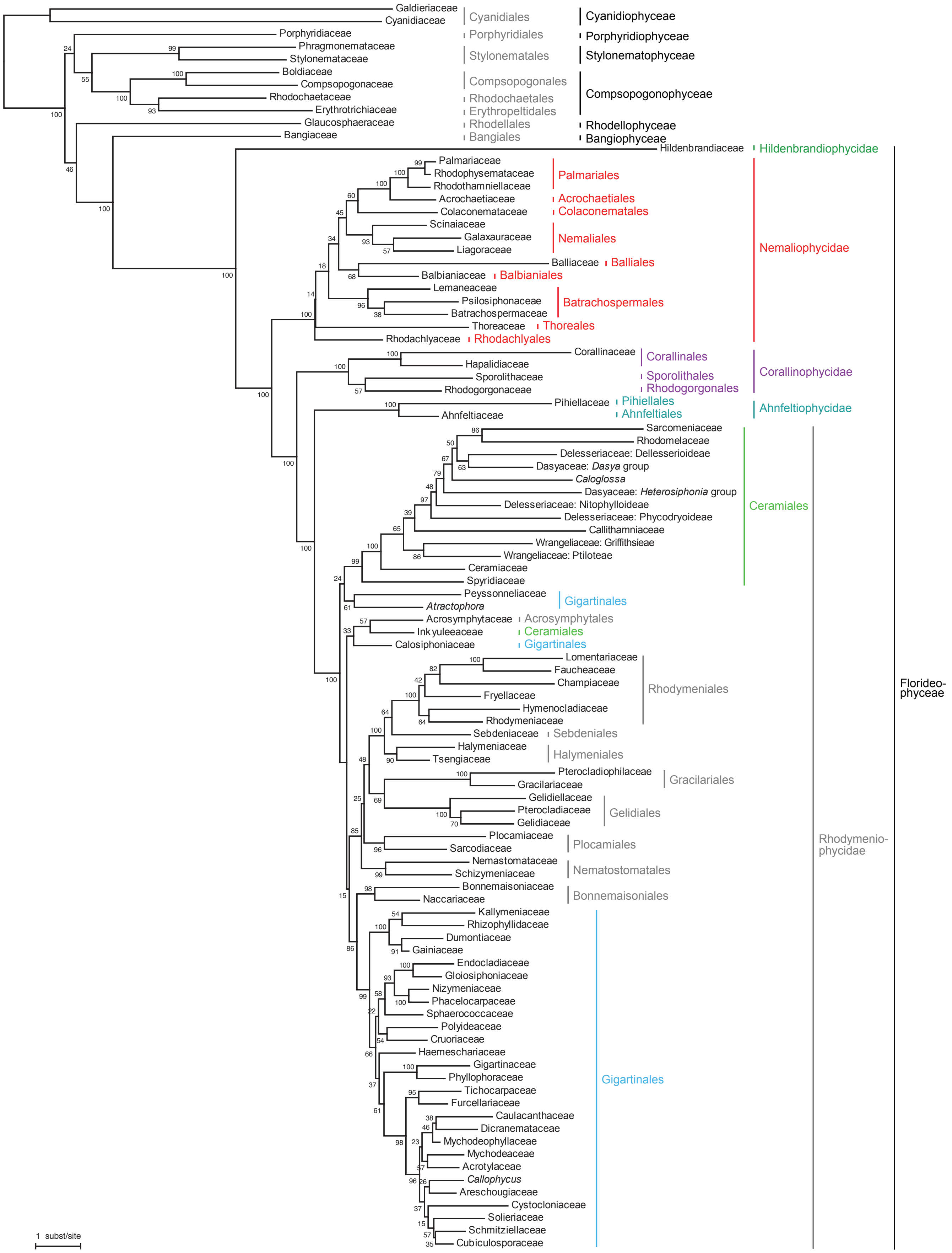

Supplement: Additional file 2 — Maximum likelihood phylogeny. Tree inferred from the 14-locus data matrix using ML inference, with ML bootstrap values at internal nodes. [file 1471-2148-10-16-S2.PDF]
