# Supplementary material for: Data mining approach identifies research priorities and data requirements for resolving the red algal tree of life
Source: BMC Evol Biol. 2010 Jan 20;10:16. doi: 10.1186/1471-2148-10-16 (PMC2826327; doi:10.1186/1471-2148-10-16)

Additional file 3. Partial data availability matrices for five poorly supported regions. Four statistics describing current data availability and the relative difficulty of resolving the region are given below the matrices (see also Table 2 in main paper). The proportion of potentially informative loci and the data overlap among potentially informative loci measure current data availability. Potentially informative loci are those that are present for more than three of the OTUs in the matrix. Data overlap is given as the average relative edge weight in the intersection graph of informative loci (see methods). The relative age and node density may indicate how difficult resolving the region will be. The relative age represents how ancient the region is, on a scale from zero (the present) to one (the root of our tree). The node density index is proportional to the number of nodes that need to be resolved per time unit (see methods).

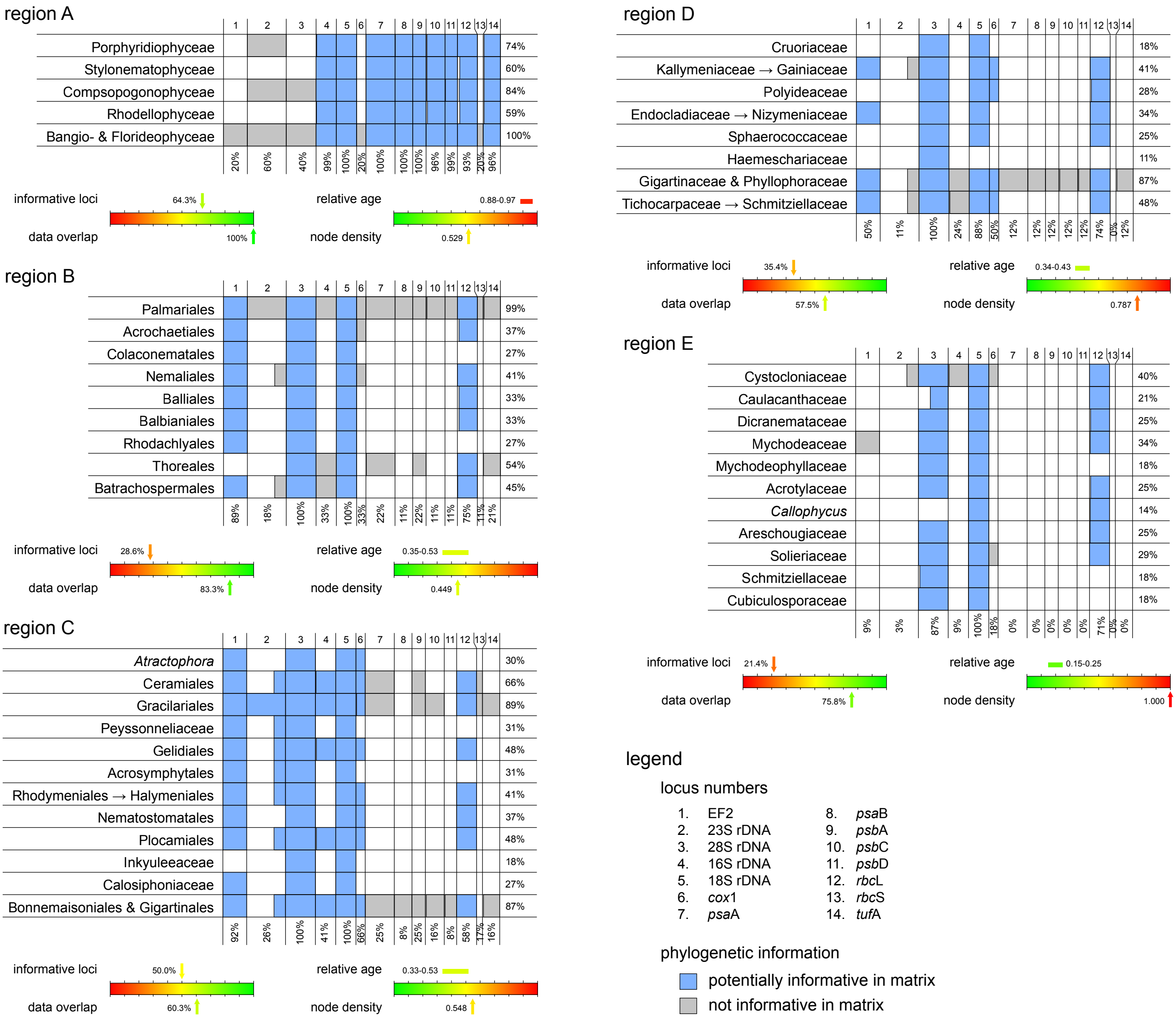

Supplement: Additional file 3 — Partial data availability matrices for five poorly supported regions. Four statistics describing current data availability and the relative difficulty of resolving the region are given below the matrices (see also Table 2 in main paper). The proportion of potentially informative loci and the data overlap among potentially informative loci measure current data availability. Potentially informative loci are those that are present for more than three of the OTUs in the matrix. Data overlap is given as the average relative edge weight in the intersection graph of informative loci (see methods). The relative age and node density may indicate how difficult resolving the region will be. The relative age represents how ancient the region is, on a scale from zero (the present) to one (the root of our tree). The node density index is proportional to the number of nodes that need to be resolved per time unit (see methods). [file 1471-2148-10-16-S3.PDF]
