# Supplementary material for: Data mining approach identifies research priorities and data requirements for resolving the red algal tree of life
Source: BMC Evol Biol. 2010 Jan 20;10:16. doi: 10.1186/1471-2148-10-16 (PMC2826327; doi:10.1186/1471-2148-10-16)

Additional file 4. Spectral partitioning. The five regional subalignments were subjected to spectral partitioning, a technique that partitions alignments based on character compatibility, the sites most compatible with each other ending up in the same partition. In order to identify potential data conflict between gene types (protein and rDNA), genomes (mitochondrial, nuclear and plastid) and individual loci, we plotted the relative contribution of each spectral partition for each gene type, genome and locus. If the relative contribution of spectral partitions differs strongly between gene types, genomes or loci, this can be taken as evidence for conflict between them. If, on the other hand, similar proportions are found, the conflict within them exceeds the conflict between them, indicating that the different gene types, genomes and loci contain similar phylogenetic signal. Note that the spectral partitions are calculated for each region separately and spectral partitions should thus not be compared between regions as any given site may have been assigned to different partitions for different regions.

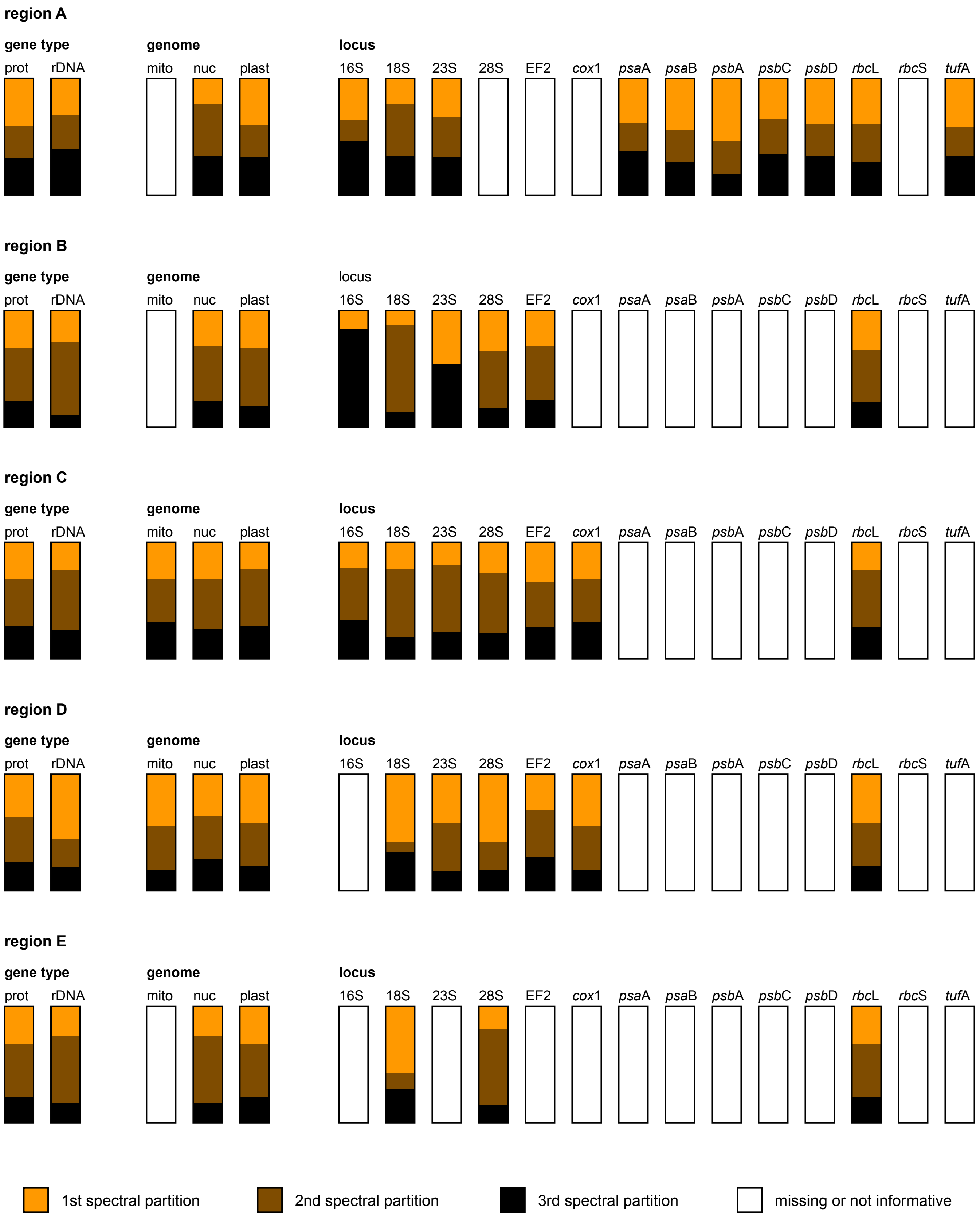

Supplement: Additional file 4 — Spectral partitioning. The five regional subalignments were subjected to spectral partitioning, a technique that partitions alignments based on character compatibility, the sites most compatible with each other ending up in the same partition. In order to identify potential data conflict between gene types (protein and rDNA), genomes (mitochondrial, nuclear and plastid) and individual loci, we plotted the relative contribution of each spectral partition for each gene type, genome and locus. If the relative contribution of spectral partitions differs strongly between gene types, genomes or loci, this can be taken as evidence for conflict between them. If, on the other hand, similar proportions are found, the conflict within them exceeds the conflict between them, indicating that the different gene types, genomes and loci contain similar phylogenetic signal. Note that the spectral partitions are calculated for each region separately and spectral partitions should thus not be compared between regions as any given site may have been assigned to different partitions for different regions. [file 1471-2148-10-16-S4.PDF]
