# Supplementary material for: Data mining approach identifies research priorities and data requirements for resolving the red algal tree of life
Source: BMC Evol Biol. 2010 Jan 20;10:16. doi: 10.1186/1471-2148-10-16 (PMC2826327; doi:10.1186/1471-2148-10-16)
Supplement: Additional file 5 — Data matrix with GenBank accession numbers. List of sequences included in our alignment, with Genbank accession numbers and the species from which they originated. [file 1471-2148-10-16-S5.PDF]

Additional file 5. Data matrix with GenBank accession numbers. List of sequences included in our alignment, with Genbank accession numbers and the species from which they originated.

family	EF2	23S rDNA	28S rDNA	16S rDNA	18S rDNA	*cox*1	*psa*A	*psa*B	*psb*A	*psb*C	*psb*D	*rbc*L	*rbc*S	*tuf*A
Acrochaetiaceae	Acrochaetium secundatum EF033523 (1682 nt)	—	Rhodochorton tenue AF421126 (2701 nt)	—	Rhodochorton tenue AF079796 (1764 nt)	Acrochaetium sp. *GQ497300 (645)	—	—	—	—	—	Audouinella arcuata AF029138 (1282 nt)	—	—
Acrosymphytaceae	Acrosymphyton caribaeum EF033539 (1680 nt)	Acrosymphyton taylorii EF426601 (362 nt)	Acrosymphyton purpuriferum DQ343683 (2842 nt)	—	Acrosymphyton purpuriferum AF317091 (1768 nt)	—	—	—	—	—	—	—	—	—
Acrotylaceae	—	—	Acrotylus sp. *GQ406346 (2692 nt)	—	Amphiplexia hymenocladioides AY437648 (1774 nt)	—	—	—	—	—	—	Hennedya crispa DQ343619 (1341 nt)	—	—
Ahnfeltiaceae	Ahnfeltia plicata EF033537 (1680 nt)	—	Ahnfeltia plicata AF419105 (2684 nt)	Ahnfeltia plicata AY731515 (1259 nt)	Ahnfeltia plicata Z14139 (1758 nt)	Ahnfeltia fastigiata *GQ497301 (664 nt)	—	—	—	—	—	Ahnfeltia plicata U04168 (1332 nt)	—	—
Areschougiaceae	—	—	Areschougia sp. *GQ406347 (2691 nt)	—	Austroclonium charoides AY437666 (1773 nt)	—	—	—	—	—	—	Erythroclonium angustatum AF099688 (1379 nt)	—	—
Atractophora	Atractophora sp. *GQ497322 (1680 nt)	—	Atractophora sp. *GQ497323 (2839 nt)	—	Atractophora hypnoides AY772728 (1777 nt)	Atractophora sp. *GQ497303 (664 nt)	—	—	—	—	—	—	—	—
Balbianiaceae	Balbiania investiens EF033524 (1650 nt)	—	Balbiania investiens AF421124 (2719 nt)	—	Rhododraparnaldia oregonica AF026043 (1764 nt)	—	—	—	—	—	—	Rhododrapamaldia oregonica AF029156 (1280 nt)	—	—
Balliaceae	Ballia callitricha EF033525 (1683 nt)	—	Ballia callitricha AF419106 (2910 nt)	—	Ballia callitricha AF236790 (1772 nt)	—	—	—	—	—	—	Ballia callitricha AF149029 (1275 nt)	—	—
Bangiaceae	Bangia atropurpurea EF033517 (1680 nt)	Porphyra purpurea U38804 (2888 nt)	Porphyra sp. EF033597 (3023 nt)	Porphyra yezoensis AP006715 (1496)	Porphyra acanthophora AY766359 (1838 nt)	Porphyra umbilicalis DQ191333 (539 nt)	Porphyra purpurea U38804 (2259 nt)	Bangia atropurpurea AY391374 (1266 nt)	Bangia atropurpurea AY119734 (954 nt)	Porphyra yezoensis AP006715 (1467 nt)	Bangia atropurpurea AY876227 (888 nt)	Porphyra dentata AB118579 (1467 nt)	Porphyra yezoensis AB118588 (417 nt)	Porphyra purpurea U38804 (1230 nt)
Batrachospermaceae	Petrohua bernabei EF033526 (1686 nt)	Batrachospermum sp. EF426659 (362 nt)	Petrohua bernabei AY960689 (2771 nt)	—	Batrachospermum macrosporum AF026048 (1763 nt)	—	—	—	—	—	—	Batrachospermum helminthosum AB114646 (1398 nt)	—	—
Boldiaceae	—	—	—	—	Boldia erythrosiphon AF055299 (1603 nt)	—	—	—	—	—	—	Boldia erythrosiphon AF087122 (1116 nt)	—	—
Bonnemaisoniaceae	Bonnemaisonia hamifera AY010232 (1680 nt)	Asparagopsis taxiformis EF426629 (362 nt)	Delisea hypneoides EF033603 (2709 nt)	Bonnemaisonia asparagoides AY731516 (1260 nt)	Asparagopsis taxiformis AY772723 (1795 nt)	—	—	—	—	—	—	Delisea pulchra U26812 (1397 nt)	—	—
Callithamniaceae	—	Euptilocladia magruderi EF426620 (361 nt)	Sciurothamnion sp. DQ022801 (1261 nt)	Sciurothamnion sp. DQ026694 (1399 nt)	Aglaothamnion callophyllidicola AY643486 (1770 nt)	—	—	—	—	—	—	Euptilota molle DQ022829 (1419 nt)	—	—
Callophycus	—	—	—	—	Callophycus oppositifolius AY437654 (1772 nt)	—	—	—	—	—	—	Callophycus oppositifolius AF099686 (1369 nt)	—	—
Caloglossa	—	—	Caloglossa axillaris AF522228 (1646 nt)	—	Caloglossa leprieurii AF488401 (1788 nt)	—	—	—	—	—	—	Caloglossa intermedia AF254164 (1419 nt)	Caloglossa continua AB023380 (150 nt)	—
Calosiphoniaceae	Schmitzia sp. EF033538 (1680 nt)	—	Schmitzia sp. DQ343685 (2718 nt)	—	Schmitzia sp. AY437659 (1761 nt)	—	—	—	—	—	—	—	—	—
Caulacanthaceae	—	—	Caulacanthus ustulatus AY920904 (874 nt)	—	Catenella caespitosa AY437661 (1788 nt)	—	—	—	—	—	—	Caulacanthus ustulatus AF321113 (1419 nt)	—	—
Ceramiaceae	Ceramium virgatum EF033544 (1680 nt)	—	Centroceras clavulatum AF419113 (2732 nt)	Antithamnionella spirographidis DQ026680 (1391 nt)	Ceramium virgatum AF236793 (1769 nt)	—	Antithamnion nipponicum AY295136 (1941 nt)	—	Antithamnion sp. X55364 (954 nt)	—	—	Centroceras minutum DQ374321 (1419 nt)	Antithamnion sp. X54532 (387 nt)	—
Champiaceae	Chylocladia verticillata EF033573 (1680 nt)	—	Chylocladia verticillata DQ343712 (2697 nt)	—	Champia affinis U23951 (1766 nt)	—	—	—	—	—	—	Champia compressa AY294358 (1419 nt)	—	—
Colaconemataceae	Colaconema caespitosum EF033528 (1680 nt)	—	Colaconema tetraspora AF528051 (2697 nt)	—	Colaconema pectinatum AF079790 (1764 nt)	—	—	—	—	—	—	—	—	—
Compsopogonaceae	—	Compsopogon caeruleus AM084276 (2735 nt)	—	Compsopogon caeruleus AF170713 (1395 nt)	Compsopogon caeruleus AF342748 (1769 nt)	—	Compsopogon caeruleus AY119701 (1395 nt)	Compsopogon caeruleus AY391375 (1266 nt)	Compsopogon caeruleus AY119737 (954 nt)	Compsopogon caeruleus AY876203 (1001 nt)	Compsopogon caeruleus AY876228 (855 nt)	Compsopogon caeruleus U04037 (1284 nt)	—	Compsopogon caeruleus AF545589 (975 nt)
Corallinaceae	Corallina officinalis EF033530 (1679 nt)	—	Amphiroa fragilissima EF033599 (2778 nt)	Corallina officinalis AY731519 (1258 nt)	Hydrolithon reinboldii DQ629000 (1934 nt)	Corallina elongata DQ191345 (539 nt)	—	—	—	—	—	Amphiroa fragilissima U04039 (1257 nt)	—	—
Cruoriaceae	—	—	Cruoria sp. *GQ406348 (2736 nt)	—	Cruoria pellita AY437664 (1777 nt)	—	—	—	—	—	—	—	—	—
Cubiculosporaceae	—	—	Cubiculosporum koronicarpus DQ343686 (2925 nt)	—	Cubiculosporum koronicarpus AY437665 (1779 nt)	—	—	—	—	—	—	—	—	—
Cyanidiaceae	Cyanidioschyzon merolae AB095183 (1695 nt)	Cyanidium caldarium AF022186 (2918 nt)	Cyanidioschyzon merolae AB158485 (3234 nt)	Cyanidium caldarium AF022186 (1527 nt)	Cyanidioschyzon merolae AF441376 (679 nt)	Cyanidioschyzon merolae D89861 (534 nt)	Cyanidioschyzon merolae AB002583 (2247 nt)	Cyanidioschyzon merolae AY391376 (1266 nt)	Cyanidioschyzon merolae AY119729 (954 nt)	Cyanidium caldarium AF022186 (1383 nt)	Cyanidium sp. AY876229 (888 nt)	Cyanidioschyzon merolae AB002583 (1467 nt)	Cyanidium caldarium Z21723 (327 nt)	Cyanidioschyzon merolae AB002583 (1233 nt)
Cystocloniaceae	—	Hypnea valentiae EF426626 (362 nt)	Hypnea charoides *GQ406354 (2713 nt)	Hypnea sp. AY731512 (1258 nt)	Fimbrifolium dichotomum AY437674 (1780 nt)	Rhodophyllis sp. AY970627 (709 nt)	—	—	—	—	—	Hypnea spinella AF385635 (1419 nt)	—	—
Dasyaceae: *Dasya* group	—	—	Dasya baillouviana AF259424 (958 nt)	—	Dasysiphonia chejuensis AF488388 (1766 nt)	Rhodoptilum plumosum *GQ497321 (664 nt)	—	—	—	—	—	Dipterocladia pinnatifolia *GQ425223 (1489 nt)	—	—
Dasyaceae: *Heterosiphonia* group	Heterosiphonia plumosa EF033546 (1679 nt)	—	Heterosiphonia plumosa EF033606 (2772 nt)	—	Thuretia quercifolia AF488400 (1792 nt)	Heterosiphonia sp. *GQ497304 (664 nt)	—	—	—	—	—	Heterosiphonia plumosa AF259494 (1361 nt)	—	—
Delesseriaceae: Delesserioideae	Grinnellia americana EF033547 (1675 nt)	—	Grinnellia americana EF033607 (2770 nt)	—	Hypoglossum hypoglossoides AF488405 (1770 nt)	—	—	—	—	—	—	Pseudophycodrys pulcherrima AF257442 (1419 nt)	—	—
Delesseriaceae: Nitophylloideae	—	Martensia fragilis EF426604 (361 nt)	Calonitophyllum medium AF259413 (1213 nt)	—	—	—	—	—	—	—	—	Augophyllum kentingii AY680694 (1419 nt)	—	—
Delesseriaceae: Phycodryoideae	Sorella repens EF033548 (1667 nt)	—	Sorella repens EF033608 (2754 nt)	—	Cryptopleura crispa AY617139 (1778 nt)	Hymenena sp. *GQ497305 (664 nt)	—	—	—	—	—	Phycodrys quercifolia AF257425 (1419 nt)	—	—
Dicranemataceae	—	—	Dicranema sp. *GQ406349 (2689 nt)	—	Dicranema revolutum AY437678 (1771 nt)	—	—	—	—	—	—	Dicranema revolutum AY294379 (1343 nt)	—	—
Dumontiaceae	Weeksia coccinea EF033552 (1680 nt)	—	Neodilsea borealis EF033610 (2750 nt)	—	Dudresnaya capricornica AF317098 (1774 nt)	Neodilsea sp. AY970616 (709 nt)	—	—	—	—	—	Dumontia contorta AY294378 (1419 nt)	—	—
Endocladiaceae	Gloiopeltis furcata EF033553 (1680 nt)	—	Gloiopeltis furcata EF033612 (2698 nt)	—	Gloiopeltis furcata U33130 (1766 nt)	—	—	—	—	—	—	Endocladia muricata U04193 (1179 nt)	—	—
Erythrotrichiaceae	—	—	—	Erythrotrichia carnea AF545619 (1349 nt)	Erythrotrichia carnea L26189 (1762 nt)	—	Erythrotrichia carnea AY119703 (1386 nt)	—	Erythrotrichia carnea AY119739 (947 nt)	—	—	Smithora naiadum AF087119 (1177 nt)	—	Erythrotrichia carnea AF545593 (975 nt)

family	EF2	23S rDNA	28S rDNA	16S rDNA	18S rDNA	*cox*1	*psa*A	*psa*B	*psb*A	*psb*C	*psb*D	*rbc*L	*rbc*S	*tuf*A
**Faucheaceae**	*Gloiocladia halymenioides*<br>EF033574 (1680 nt)	—	*Gloiocladia repens*<br>AF419143 (2684 nt)	—	*Webervanbossea splachnoides*<br>AF085269 (1761 nt)	*Leptofauchea pacifica*<br>AY970581 (709 nt)	—	—	—	—	—	*Gloiocladia laciniata*<br>AY294355 (1419 nt)	—	—
**Fryeellaceae**	*Fryeella gardneri*<br>EF033578 (1679 nt)	—	*Fryeella gardneri*<br>EF033622 (2700 nt)	—	*Fryeella gardneri*<br>AF085273 (1762 nt)	*Fryeella* sp.<br>*GQ497306 (664 nt)	—	—	—	—	—	*Hymenocladiopsis crustigena*<br>U21640 (974 nt)	—	—
**Furcellariaceae**	—	—	*Furcellaria* sp.<br>*GQ406350 (2686 nt)	—	*Furcellaria lumbricalis*<br>Z14141 (1770 nt)	*Opuntiella californica*<br>*GQ497302 (664 nt)	—	—	—	—	—	*Furcellaria lumbricalis*<br>AY294371 (1374 nt)	—	—
**Gainiaceae**	—	—	*Gainia* sp.<br>*GQ406351 (2749 nt)	—	*Gainia mollis*<br>AF317107 (1772 nt)	—	—	—	—	—	—	—	—	—
**Galaxauraceae**	—	*Dichotomaria marginata*<br>EF426656 (362 nt)	*Dichotomaria diesingiana*<br>AY570364 (2824 nt)	—	—	—	—	—	—	—	—	*Galaxaura pacifica*<br>AY688004 (1419 nt)	—	—
**Galdieriaceae**	—	—	—	*Galdieria sulphuraria*<br>AF170718 (1394 nt)	*Galdieria* sp.<br>AY343928 (1801 nt)	—	*Galdieria sulphuraria*<br>AY119696 (1395 nt)	*Galdieria sulphuraria*<br>AY391381 (1266 nt)	*Galdieria sulphuraria*<br>AY119731 (954 nt)	*Galdieria sulphuraria*<br>AY876207 (1002 nt)	*Galdieria sulphuraria*<br>AY876232 (882 nt)	*Galdieria sulphuraria*<br>X55524 (1482 nt)	*Galdieria sulphuraria*<br>X55524 (417 nt)	*Galdieria sulphuraria*<br>AF545591 (975 nt)
**Gelidiaceae**	*Gelidium australe*<br>EF033549 (1680 nt)	*Gelidium reediae*<br>EF426619 (362 nt)	*Gelidium australe*<br>DQ343682 (2701 nt)	*Gelidium caulacantheum*<br>AY706944 (1356 nt)	*Gelidium amansii*<br>DQ316994 (1766 nt)	*Gelidium purpurascens*<br>*GQ497307 (664 nt)	—	—	—	—	—	*Gelidium vittatum*<br>AF501289 (1419 nt)	—	—
**Gelidiellaceae**	—	—	*Gelidiella acerosa*<br>AF039551 (2510 nt)	*Gelidiella acerosa*<br>AY706939 (1354 nt)	*Gelidiella ligulata*<br>AB017669 (1685 nt)	—	—	—	—	—	—	*Parviphycus tenuissimus*<br>AF320983 (1419 nt)	—	—
**Gigartinaceae**	*Chondrus crispus*<br>EF033554 (1680 nt)	—	*Chondrus crispus*<br>AF419120 (2679 nt)	*Chondrus crispus*<br>Z29521 (1391 nt)	*Chondrus crispus*<br>DQ316999 (1776 nt)	*Mazzaella rosea*<br>AY970600 (709 nt)	*Chondrus crispus*<br>AY119710 (1395 nt)	*Chondrus crispus*<br>AY391390 (543 nt)	*Chondrus crispus*<br>AY119746 (934 nt)	*Chondrus crispus*<br>AY876210 (990 nt)	*Chondrus crispus*<br>AY876235 (821 nt)	*Chondracanthus saundersii*<br>AY698062 (1419 nt)	—	*Chondrus crispus*<br>AF545602 (975 nt)
**Glaucosphaeraceae**	—	—	—	*Dixoniella grisea*<br>AF545621 (1356 nt)	*Rhodella cyanea*<br>AB045605 (1756 nt)	—	*Dixoniella grisea*<br>AY119702 (1395 nt)	*Dixoniella grisea*<br>AY391383 (1266 nt)	*Rhodella violacea*<br>AY119742 (954 nt)	*Rhodella violacea*<br>DQ308461 (933 nt)	*Rhodella violacea*<br>DQ308462 (888 nt)	*Rhodella violacea*<br>AY119776 (1215 nt)	—	*Dixoniella grisea*<br>AF545595 (975 nt)
**Gloiosiphoniaceae**	—	—	*Gloiosiphonia* sp.<br>*GQ406352 (2697 nt)	—	*Gloiosiphonia capillaris*<br>AY437680 (1770 nt)	—	—	—	—	—	—	*Gloiosiphonia verticillata*<br>U04195 (1236 nt)	—	—
**Gracilariaceae**	*Gracilaria salicornia*<br>EF033559 (1680 nt)	*Gracilaria tenuistipitata*<br>AY673996 (2888 nt)	*Gracilariopsis andersonii*<br>AF419132 (2807 nt)	*Gracilaria tenuistipitata*<br>AY673996 (1494 nt)	*Gracilariophila oryzoides*<br>U43556 (1780 nt)	*Gracilaria gracilis*<br>DQ191346 (539 nt)	*Gracilaria tenuistipitata*<br>AY673996 (2301 nt)	—	*Gracilaria tenuistipitata*<br>AY673996 (1083 nt)	*Gracilaria tenuistipitata*<br>AY673996 (1461 nt)	—	*Gracilaria tenuistipitata*<br>AY673996 (1467 nt)	*Gracilaria tenuistipitata*<br>AY673996 (417 nt)	*Gracilaria tenuistipitata*<br>AY673996 (1230 nt)
**Haemeschariaceae**	—	—	*Haemescharia* sp.<br>*GQ406353 (2714 nt)	—	—	—	—	—	—	—	—	—	—	—
**Halymeniaceae**	*Isabbottia ovalifolia*<br>EF033563 (1680 nt)	*Polyopes hakalauensis*<br>EF426602 (362 nt)	*Grateloupia lyallii*<br>EF033617 (2769 nt)	—	*Norrissia setchellii*<br>AY437703 (1768 nt)	*Grateloupia* sp.<br>*GQ497308 (664 bp)	—	—	—	—	—	*Cryptonemia borealis*<br>AF488812 (1419 nt)	—	—
**Hapalidiaceae**	*Synarthrophyton patena*<br>EF033531 (1680 nt)	—	*Synarthrophyton patena*<br>EF033600 (2756 nt)	—	*Mesophyllum erubescens*<br>DQ629011 (1782 nt)	—	—	—	—	—	—	—	—	—
**Hildenbrandiaceae**	*Hildenbrandia rubra*<br>EF033522 (1680 nt)	*Hildenbrandia angolensis*<br>EF426600 (362 nt)	*Apophlaea lyallii*<br>AF419135 (2949 nt)	—	*Apophlaea lyallii*<br>AF076996 (1558 nt)	*Hildenbrandia rubra*<br>*GQ497309 (664 nt)	—	—	—	—	—	*Hildenbrandia angolensis*<br>AF207832 (1172 nt)	—	—
**Hymenocladiaceae**	*Hymenocladia chondricola*<br>EU624202 (1680 nt)	—	*Asteromenia pseudocoalescens*<br>DQ068298 (2794 nt)	—	*Hymenocladia chondricola*<br>AF117128 (1763 nt)	*Asteromenia pseudocoalescens*<br>AY970629 (664 nt)	—	—	—	—	—	—	—	—
**Inkyuleeaceae**	—	—	*Inkyuleea mariana*<br>DQ343681 (2733 nt)	—	*Inkyuleea ballioides*<br>AF236789 (1772 nt)	—	—	—	—	—	—	—	—	—
**Kallymeniaceae**	*Pugetia fragilissima*<br>EF033556 (1680 nt)	—	*Kallymenia cribrosa*<br>AY171611 (2716 nt)	—	*Kallymenia tasmanica*<br>AF317111 (1770 nt)	—	—	—	—	—	—	*Erythrophyllum delesserioides*<br>AF212186 (1419 nt)	—	—
**Lemaneaceae**	—	—	*Lemanea fluviatilis*<br>AF419110 (2684 nt)	*Paralemanea* sp.<br>DQ917828 (1365 nt)	*Lemanea fluviatilis*<br>AY495976 (811 nt)	—	—	—	—	—	—	*Paralemanea grandis*<br>DQ523259 (1383 nt)	—	—
**Liagoraceae**	*Nemalion helminthoides*<br>EF033532 (1686 nt)	*Ganonema farinosum*<br>EF426637 (360 nt)	*Helminthocladia australis*<br>AY570372 (2696 nt)	—	*Nemalion helminthoides*<br>L26196 (1763 nt)	*Nemalion* sp.<br>*GQ497310 (664 nt)	—	—	—	—	—	*Izziella orientalis*<br>AY688028 (677 nt)	—	—
**Lomentariaceae**	*Gelidiopsis intricata*<br>EF033575 (1679 nt)	—	*Gelidiopsis intricata*<br>EF033620 (2714 nt)	—	*Ceratodictyon spongiosum*<br>AF117127 (1775 nt)	*Lomentaria* sp.<br>*GQ497311 (664 nt)	—	—	—	—	—	*Ceratodictyon spongiosum*<br>AY294357 (1419 nt)	—	—
**Mychodeaceae**	*Mychodea acanthymenia*<br>EF033557 (1679 nt)	—	*Mychodea acanthymenia*<br>EF033614 (2700 nt)	—	*Mychodea marginifera*<br>AY437685 (1773 nt)	—	—	—	—	—	—	*Mychodea hamata*<br>AF212191 (1368 nt)	—	—
**Mychodeophyllaceae**	—	—	*Mychodeophyllum* sp.<br>*GQ406355 (2685 nt)	—	*Mychodeophyllum papillitectum*<br>AY437687 (1772 nt)	—	—	—	—	—	—	—	—	—
**Naccariaceae**	*Reticulocaulis mucosissimus*<br>EF033542 (1680 nt)	—	*Reticulocaulis mucosissimus*<br>DQ343680 (2708 nt)	—	*Naccaria wiggii*<br>AY772729 (1773 nt)	*Naccaria* sp.<br>*GQ497312 (664 nt)	—	—	—	—	—	—	—	—
**Nemastomataceae**	*Predaea kraftiana*<br>EF033567 (1680 nt)	—	*Adelophycus corneus*<br>DQ343704 (2789 nt)	—	*Predaea weldii*<br>AF515298 (1768 nt)	—	—	—	—	—	—	*Predaea feldmannii*<br>AY294366 (1419 nt)	—	—
**Nizymeniaceae**	—	—	*Nizymenia* sp.<br>*GQ406356 (2696 nt)	—	*Nizymenia australis*<br>U09616 (1772 nt)	—	—	—	—	—	—	*Nizymenia australis*<br>AF212192 (1416 nt)	—	—
**Palmariaceae**	*Palmaria palmata*<br>EF033533 (1686 nt)	*Palmaria palmata*<br>Z18289 (2885 nt)	*Palmaria palmata*<br>Y11506 (2701 nt)	*Palmaria palmata*<br>Z18289 (1486 nt)	*Palmaria palmata*<br>Z14142 (1764 nt)	*Palmaria palmata*<br>*GQ497313 (664 nt)	*Palmaria palmata*<br>AY119711 (1395 nt)	*Palmaria palmata*<br>AY391391 (1266 nt)	*Palmaria palmata*<br>AF530400 (954 nt)	*Palmaria palmata*<br>AY876211 (978 nt)	*Palmaria palmata*<br>AY876236 (888 nt)	*Palmaria palmata*<br>U28421 (1467 nt)	*Palmaria palmata*<br>U28421 (417 nt)	*Palmaria palmata*<br>AF545603 (975 nt)
**Peyssonneliaceae**	*Sonderopelta coriacea*<br>EF033558 (1680 nt)	*Peyssonnelia inamoena*<br>EF426622 (361 nt)	*Peyssonnelia* sp.<br>AF419125 (2856 nt)	—	*Peyssonnelia rubra*<br>DQ629016 (1781 nt)	—	—	—	—	—	—	—	—	—
**Phacelocarpaceae**	—	—	*Phacelocarpus* sp.<br>*GQ406357 (2695 nt)	—	*Phacelocarpus peperocarpos*<br>U09617 (1773 nt)	—	—	—	—	—	—	*Phacelocarpus tortuosus*<br>AY294372 (1419 nt)	—	—
**Phragmonemataceae**	—	—	—	—	—	—	*Kyliniella latvica*<br>DQ308451 (1398 nt)	—	—	—	—	*Kyliniella latvica*<br>DQ308431 (1215 nt)	—	—
**Phyllophoraceae**	—	*Ahnfeltiopsis concinna*<br>EF426627 (362 nt)	*Mastocarpus stellatus*<br>AF427518 (2691 nt)	—	*Stenogramme interrupta*<br>AY437691 (1773 nt)	*Mastocarpus stellatus*<br>DQ191349 (539 nt)	—	—	—	—	—	*Gymnogongrus* sp.<br>AY135170 (1419 nt)	—	—
**Pihiellaceae**	—	—	—	—	*Pihiella liagoraciphila*<br>AY301992 (1656 nt)	—	—	—	—	—	—	—	—	—
**Plocamiaceae**	*Plocamium maggsiae*<br>EF033570 (1680 nt)	*Plocamium sandvicense*<br>EF426621 (362 nt)	*Plocamium angustum*<br>AY881711 (2797 nt)	*Plocamium cartilagineum*<br>AY731513 (1258 nt)	*Plocamiocolax pulvinata*<br>U43552 (1768 nt)	*Plocamium* sp.<br>*GQ497314 (664 nt)	—	—	—	—	—	*Plocamium coleorhiza*<br>U21700 (1307 nt)	—	—
**Polyideaceae**	—	—	*Polyides* sp.<br>FJ848972 (2699 nt)	—	*Polyides rotundus*<br>AF317117 (1774 nt)	*Polyides* sp.<br>*GQ497315 (664 nt)	—	—	—	—	—	*Polyides rotundus*<br>U04214 (964 nt)	—	—
**Porphyridiaceae**	—	*Porphyridium aerugineum*<br>AM084277 (1389 nt)	—	*Porphyridium aerugineum*<br>AM084277 (1389 nt)	*Flintiella sanguinaria*<br>AF342749 (1750 nt)	—	*Porphyridium sordidum*<br>DQ308460 (1395 nt)	*Flintiella sanguinaria*<br>AY391384 (1242 nt)	*Porphyridium aerugineum*<br>AY119741 (954 nt)	*Porphyridium aerugineum*<br>AY876209 (969 nt)	*Flintiella sanguinaria*<br>DQ308464 (888 nt)	*Porphyridium aerugineum*<br>X17597 (1419 nt)	—	*Porphyridium aerugineum*<br>AF545597 (975 nt)
**Psilosiphonaceae**	—	—	—	—	*Psilosiphon scoparium*<br>AF026041 (1758 nt)	—	—	—	—	—	—	*Psilosiphon scoparum*<br>AF029155 (1282 nt)	—	—

family	EF2	23S rDNA	28S rDNA	16S rDNA	18S rDNA	*cox*1	*psa*A	*psa*B	*psb*A	*psb*C	*psb*D	*rbc*L	*rbc*S	*tuf*A
**Pterocladiaceae**	—	—	*Pterocladia lucida* AF419118 (2668 nt)	*Pterocladiella melanoidea* AY706942 (1353 nt)	*Pterocladiella capillacea* Y11957 (1720 nt)	—	—	—	—	—	—	*Pterocladiella bartlettii* AF305806 (1419 nt)	—	—
**Pterocladiophilaceae**	—	—	—	—	*Holmsella pachyderma* AY617134 (1789 nt)	—	—	—	—	—	—	—	—	—
**Rhizophyllidaceae**	—	*Portieria hornemannii* EF426603 (362 nt)	*Portieria* sp. FJ848973 (2765 nt)	—	*Portieria hornemannii* AF317118 (1775 nt)	*Portieria* sp. *GQ425224 (642 nt)	—	—	—	—	—	*Portieria hornemannii* AF212185 (1381 nt)	—	—
**Rhodachlyaceae**	*Rhodachlya madagascarensis* EU262262 (1709 nt)	—	*Rhodachlya madagascarensis* EU262261 (2665 nt)	—	*Rhodachlya madagascarensis* EU262260 (1768 nt)	—	—	—	—	—	—	—	—	—
**Rhodochaetaceae**	—	—	*Rhodochaete* sp. *GQ406358 (2741 nt)	*Rhodochaete parvula* AF545623 (1347 nt)	*Rhodochaete parvula* AF139462 (1725 nt)	—	*Rhodochaete parvula* AY119707 (776 nt)	*Rhodochaete parvula* AY391389 (1254 nt)	*Rhodochaete parvula* AY119743 (954 nt)	—	—	*Rhodochaete parvula* AY119777 (1215 nt)	—	*Rhodochaete parvula* AF545601 (975 nt)
**Rhodogorgonaceae**	*Renouxia* sp. EF033534 (1680 nt)	—	*Rhodogorgon ramosissima* EF033602 (2985 nt)	—	*Renouxia* sp. EF033584 (1894 nt)	*Renouxia* sp. *GQ497316 (653 nt)	—	—	—	—	—	*Rhodogorgon carriebowensis* U04183 (1410 nt)	—	—
**Rhodomelaceae**	—	*Acanthophora spicifera* EF426609 (361 nt)	*Cladhymenia lyallii* AF259417 (1140 nt)	*Neosiphonia harveyi* AY731510 (1256 nt)	*Leachiella pacifica* AY617133 (2257 nt)	*Rhodomela confervoides* *GQ497317 (664 nt)	—	—	—	—	—	*Chondrophycus flagellifera* AF465804 (1419 nt)	—	—
**Rhodophysemataceae**	—	—	*Rhodophysema elegans* AF419140 (2678 nt)	—	*Rhodophysema elegans* U23817 (1764 nt)	—	—	—	—	—	—	—	—	—
**Rhodothamniellaceae**	—	—	*Rhodothamniella floridula* AF420252 (2705 nt)	—	*Camontagnea oxyclada* AF079794 (1764 nt)	—	—	—	—	—	—	—	—	—
**Rhodymeniaceae**	*Cephalocystis furcellata* EF033577 (1680 nt)	*Botryocladia skottsbergii* EF426610 (361 nt)	*Irvinea ardreana* DQ343714 (2700 nt)	—	*Leptosomia rosea* AF085260 (1770 nt)	*Halichrysis* sp. AY970628 (709 nt)	—	—	—	—	—	*Rhodymenia capensis* AF385646 (1419 nt)	—	—
**Sarcodiaceae**	*Sarcodia ciliata* EF033572 (1680 nt)	—	*Sarcodia ciliata* DQ343708 (3011 nt)	—	*Sarcodia marginata* DQ343667 (1768 nt)	—	—	—	—	—	—	*Trematocarpus dichotomus* U26814 (1419 nt)	—	—
**Sarcomeniaceae**	—	—	*Sarcomenia delesserioides* AF259479 (858 nt)	—	*Platysiphonia victoriae* AF488408 (1816 nt)	—	—	—	—	—	—	*Sarcomenia delesserioides* AF257443 (1408 nt)	—	—
**Schizymeniaceae**	*Platoma cyclocolpa* EF033568 (1680 nt)	—	*Titanophora weberae* DQ343707 (2901 nt)	—	*Titanophora weberae* AF515301 (1784 nt)	*Schizymenia pacifica* *GQ497319 (664 nt)	—	—	—	—	—	*Schizymenia* sp. AY294390 (1419 nt)	—	—
**Schmitziellaceae**	—	—	*Schmitziella* sp. *GQ406359 (1642 nt)	—	*Schmitziella endophloea* AY437697 (1772 nt)	—	—	—	—	—	—	—	—	—
**Scinaiaceae**	—	—	*Nothogenia fastigiata* AY570379 (2705 nt)	—	—	*Scinaia confusa* *GQ497320 (664 nt)	—	—	—	—	—	*Scinaia okamurae* AB258450 (1379 nt)	—	—
**Sebdeniaceae**	*Sebdenia flabellata* EF033579 (1680 nt)	—	*Lesleigha* sp. DQ343699 (2800 nt)	—	*Crassitegula walsinghamia* AY964057 (1767 nt)	—	—	—	—	—	—	*Sebdenia integra* AY294363 (1419 nt)	—	—
**Solieriaceae**	—	—	*Solieria* sp. *GQ406360 (2737 nt)	—	*Betaphycus speciosum* AY437653 (1779 nt)	*Sarcodiotheca furcata* *GQ497318 (664 nt)	—	—	—	—	—	*Meristotheca papulosa* AF099700 (1419 nt)	—	—
**Sphaerococcaceae**	—	—	*Sphaerococcus* sp. FJ848974 (2696 nt)	—	*Sphaerococcus coronopifolius* U09622 (1772 nt)	—	—	—	—	—	—	*Sphaerococcus coronopifolius* AY294376 (1419 nt)	—	—
**Sporolithaceae**	—	—	*Sporolithon* sp. GQ149068 (2989 nt)	—	*Sporolithon ptychoides* DQ629014 (1850 nt)	—	—	—	—	—	—	—	—	—
**Spyridiaceae**	—	—	*Spyridia dasyoides* AF419115 (2674 nt)	—	*Spyridia dasyoides* DQ343659 (1761 nt)	—	—	—	—	—	—	*Spyridia filamentosa* AF458703 (1301 nt)	—	—
**Stylonemataceae**	—	—	—	*Chroodactylon ornatum* AF170712 (1393 nt)	*Bangiopsis* sp. AY766363 (1786 nt)	—	*Rhodosorus* sp. DQ308454 (1398 nt)	*Bangiopsis subsimplex* AY391382 (1266 nt)	*Rhodosorus marinus* AY119744 (954 nt)	*Rhodosorus marinus* AY876208 (1002 nt)	*Rhodosorus marinus* AY876233 (880 nt)	*Chroodactylon ornatum* DQ308429 (1215 nt)	—	*Bangiopsis subsimplex* AF545594 (975 nt)
**Thoreaceae**	—	—	*Thorea violacea* AF419146 (3400 nt)	*Thorea violacea* AF170721 (1396 nt)	*Thorea violacea* AF342744 (1914 nt)	—	*Thorea violacea* AY119712 (1374 nt)	—	*Thorea violacea* AY119747 (954 nt)	—	—	*Thorea hispida* AB159652 (1419 nt)	—	*Nemalionopsis shawii* AB159668 (1104 nt)
**Tichocarpaceae**	—	—	—	—	*Tichocarpus crinitus* AY437698 (1772 nt)	—	—	—	—	—	—	*Tichocarpus crinitus* U21590 (1271 nt)	—	—
**Tsengiaceae**	—	—	*Tsengia comosa* DQ343702 (2701 nt)	—	*Tsengia laingii* AF515302 (1765 nt)	—	—	—	—	—	—	*Tsengia lanceolata* AY294386 (1419 nt)	—	—
**Wrangeliaceae: Griffithsieae**	—	—	—	—	*Anotrichium furcellatum* U32561 (1765 nt)	—	*Anotrichium yagii* AY295123 (1938 nt)	—	*Griffithsia traversii* AY295154 (939 nt)	—	—	*Griffithsia corallinoides* AY295164 (1419 nt)	—	—
**Wrangeliaceae: Ptiloteae**	*Ptilota serrata* EF033545 (1680 nt)	—	*Ptilota serrata* EF033605 (2745 nt)	*Ptilota serrata* DQ026676 (1390 nt)	*Ptilota serrata* AF203884 (1761 nt)	*Ptilota serrata* AY970640 (709 nt)	—	—	*Plumaria plumosa* AY865139 (894 nt)	—	—	*Plumaria plumosa* DQ022808 (1419 nt)	—	—
